# Elucidating ionic–electronic transport in cation-disordered rocksalt cathodes

**DOI:** 10.1039/d6sc06335g

**Published:** 2026-09-18

**Authors:** Miaomiao Wang, Yuyang Zhang, Zhanhui Jia, Hao Shen, Wei Tang, Kai Chen

**Affiliations:** a Center for Advancing Materials Performance from the Nanoscale (CAMPNano), State Key Laboratory for Mechanical Behavior of Materials, Xi'an Jiaotong University Xi'an Shaanxi 710049 P. R. China jiazhanhui8@163.com kchenlbl@gmail.com; b Suzhou National Laboratory Suzhou Jiangsu 215123 P. R. China shenh@szlab.ac.cn; c School of Chemical Engineering and Technology, Xi'an Jiaotong University Xi'an Shaanxi 710049 P. R. China

## Abstract

High-capacity cation-disordered rocksalt (DRX) cathodes have created new opportunities for low-cost, high-energy-density lithium-ion batteries. By combining cationic and anionic redox reactions, Li-excess DRX cathodes can deliver capacities beyond those of conventional layered oxides. However, their practical implementation is still constrained by sluggish Li^+^ transport, intrinsically poor electronic conductivity, severe interfacial instability at high voltages, as well as the frequent demands for nanosized particles and large fractions of conductive carbon. In this review, we critically summarize recent progress in understanding ionic–electronic transport in DRX cathodes. We first discuss Li^+^ migration through 0-TM percolation networks, the influence of short-range ordering (SRO) and heterogeneous Li-site energy landscapes, and strategies for enhancing ionic transport through SRO regulation, δ-phase formation, partial disordering, and activation of additional diffusion pathways. Then electronic transport mechanisms are analyzed based on localized small-polaron hopping, and the electronic-structure and electrode-level approaches are reviewed for improving conductivity. Finally, design principles are proposed that balance Li^+^ percolation, electronic conduction, d^0^ transition-metal selection, local ordering, and interfacial stability, and future directions for scalable synthesis, electrolyte/interface engineering, all-solid-state batteries, advanced characterization, and data-driven materials discovery are outlined.

## Abstract 2

This review summarizes the ionic–electronic transport mechanisms of DRX cathodes, highlights the need to simultaneously optimize Li^+^ percolation and electronic conductivity, and proposes design strategies for high-performance DRX cathodes.

## Graphical abstract

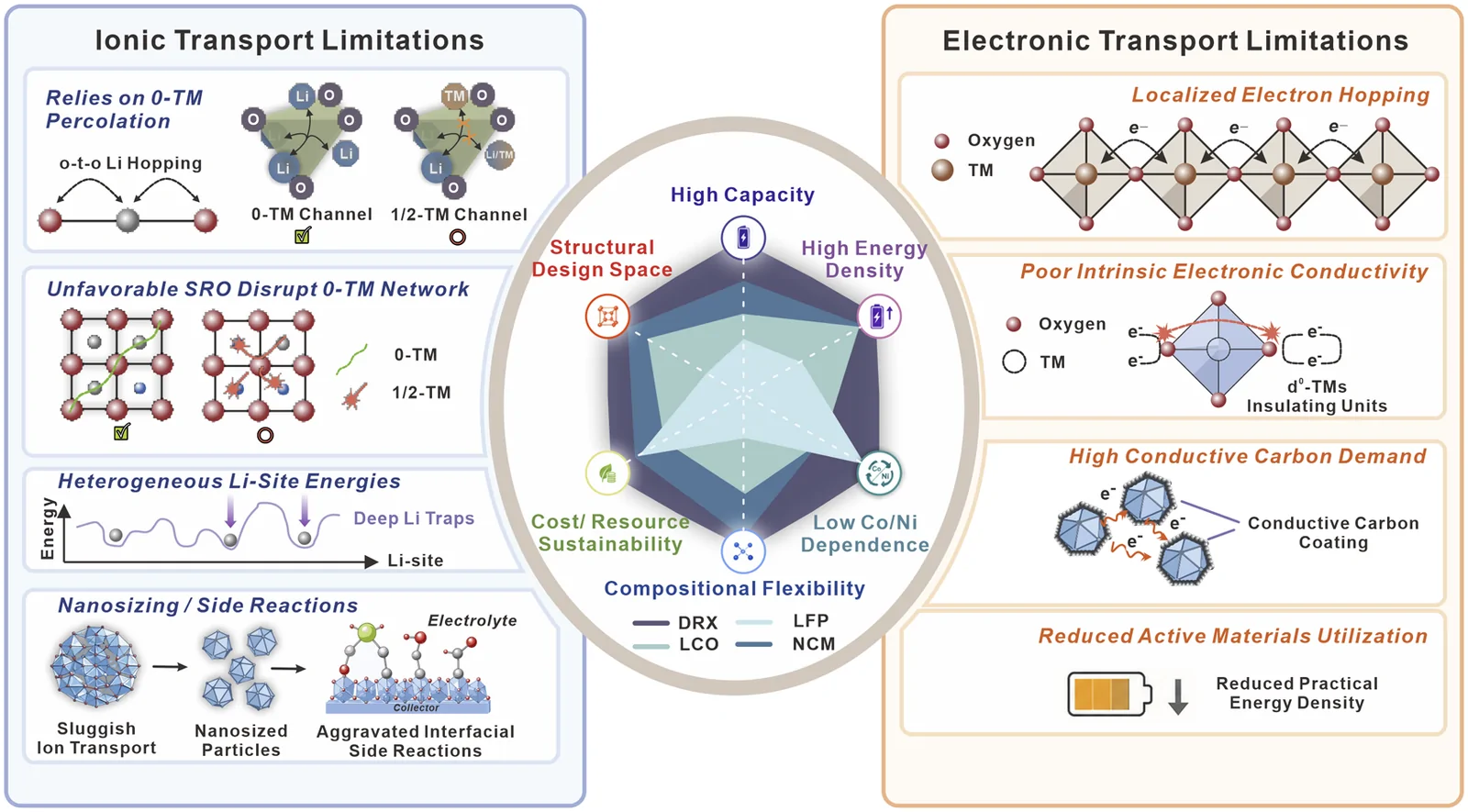

## 1 Introduction

Driven by the increasing demands for high-energy-density electrochemical storage, rechargeable lithium-ion batteries (LIBs) have become indispensable for portable electronics, electric vehicles, and large-scale grid applications because of their high energy density, long cycle life, and high energy-conversion efficiency.^1,2^ However, conventional layered cathode materials face persistent challenges associated with the high cost, limited abundance, and structural instability of Co- and Ni-rich compositions, especially under high-voltage operation.^3,4^ These limitations have motivated extensive efforts to develop next-generation cathode materials that combine high capacity, improved sustainability, and low cost.

Cation-disordered rocksalt (DRX) cathodes have attracted considerable attention in this context. Their Li-excess disordered lattices enable concurrent cationic and anionic redox reactions, allowing capacities substantially higher than those of conventional layered oxides.^5,6^ Layered cathodes rely on well-ordered lithium diffusion channels, whereas DRX materials are based on LiMO_2_-type frameworks in which Li and transition-metal (TM) cations are randomly distributed.^7^ When the Li content exceeds that of the TM sublattice, excess Li^+^ can occupy octahedral sites within TM layers. Materials with partial Li/TM segregation are usually classified as Li-rich layered oxides, whereas DRX cathodes exhibit random Li/TM distribution throughout the lattice without obvious layered segregation and can generally be described as Li_1+*x*_TM_1−*x*_O_2_.^8–10^

**Fig. 1:**
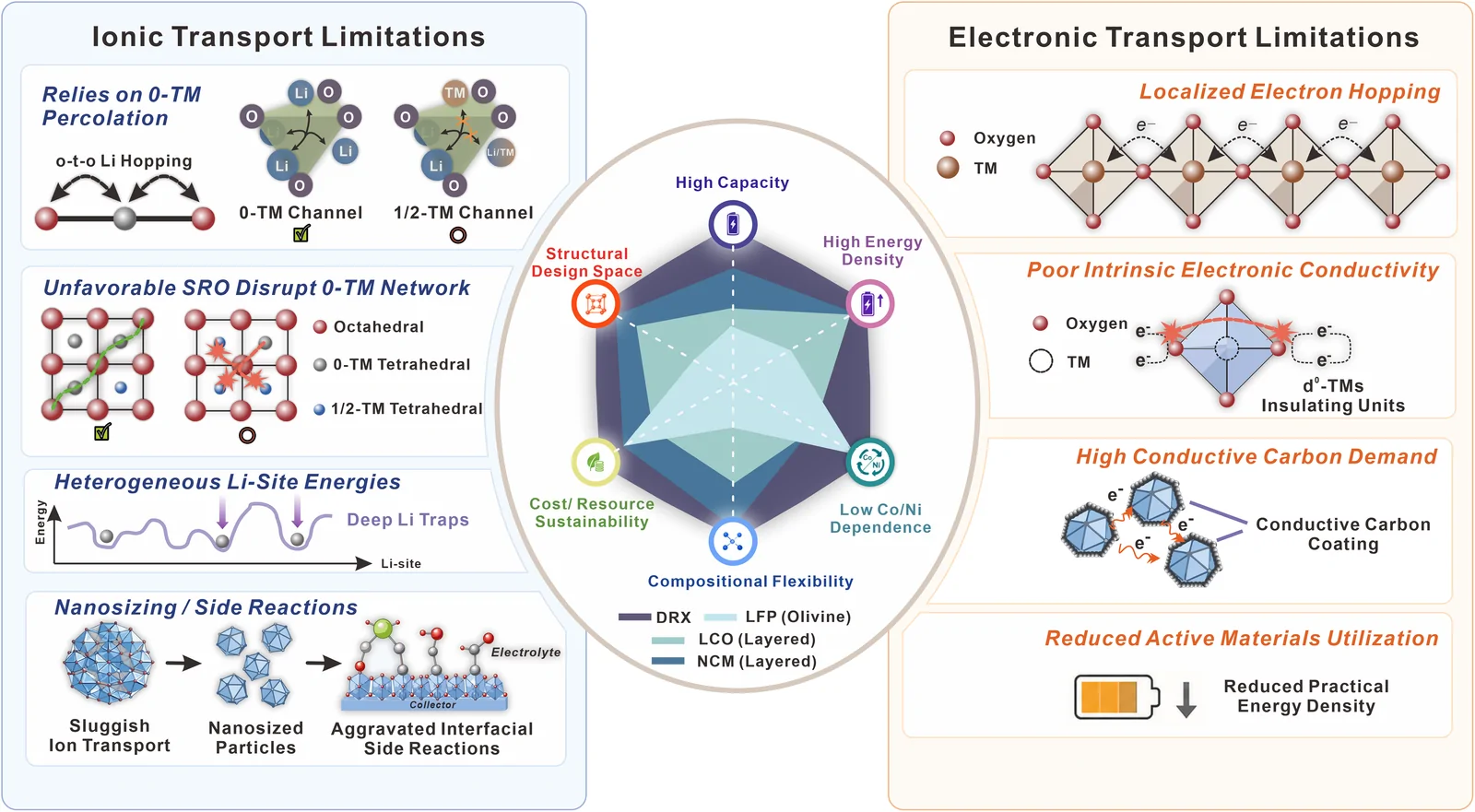
Origin of sluggish ionic and electronic transport in DRX cathodes, with a comparison between DRX and typical cathode materials, such as lithium nickel cobalt manganese oxide (NCM), lithium iron phosphate (LFP), and lithium cobalt oxide (LCO), inserted in the middle.

Nevertheless, sluggish charge-transport kinetics remain a central obstacle to the practical application of DRX cathodes (Fig. 1).^11^ For ionic transport, Li^+^ migration in DRX materials depends strongly on interconnected low-barrier 0-TM diffusion channels, in which no TM cations face-share the intermediate tetrahedral site.^12,13^ The continuity of these channels can be disrupted by unfavorable short-range ordering (SRO), thereby limiting the utilization of electrochemically active Li-storage sites. Unlike the relatively ordered two-dimensional Li^+^ diffusion pathways in layered oxides, DRX cathodes contain highly heterogeneous and tortuous diffusion routes arising from random cation distributions. As a result, reported Li^+^ diffusivities in DRX cathodes typically range from 10^−14^ to 10^−17^ cm^2^ s^−1^, several orders of magnitude lower than those of typical layered cathodes (10^−9^ to 10^−12^ cm^2^ s^−1^).^14^ To compensate for this sluggish transport, high-performance DRX cathodes often require nanosized particles (<500 nm) to shorten diffusion lengths and enhance Li^+^ accessibility.^15–17^ However, nanosizing increases surface area, accelerates parasitic electrolyte reactions, and often relies on energy-intensive ball milling, creating challenges for scalable manufacturing. Enabling fast Li^+^ migration in micrometer-sized particles is therefore essential for commercializing low-cost DRX cathodes.

Compounding their sluggish ionic transport, the intrinsically poor electronic conductivity of DRX materials severely bottlenecks electrochemical performance. Strong electron localization within the disordered lattice leads to electronic transport dominated by small-polaron hopping, resulting in low intrinsic conductivity.^18^ Consequently, most DRX electrodes require high conductive-carbon contents, typically 20–30 wt%, to compensate for inadequate electron transport.^19^ Such high carbon fractions reduce practical electrode energy density and are incompatible with commercial cathode formulations, in which conductive additives are generally limited to below 5 wt%. Improving intrinsic and electrode-level electronic conductivity is therefore equally important for the practical implementation of DRX cathodes.

Recent studies have substantially advanced the understanding of how local cation configurations, electronic structures, and interfacial chemistry regulate Li^+^ diffusion and electronic transport in DRX systems. However, existing reviews and individual studies remain fragmented and have often focused more on anionic-redox controllability, reversibility, and voltage hysteresis than on coupled ionic–electronic transport. Herein, we systematically elucidate the fundamental mechanisms governing Li^+^ migration and electronic conduction in DRX cathodes, while comprehensively reviewing recent breakthroughs in charge-transport optimization strategies. Furthermore, the pivotal roles of advanced characterization techniques and theoretical simulations are highlighted. Building upon these insights, we outline rational design principles to realize high-rate, high-energy-density, and commercially viable DRX architectures. Ultimately, this review aims to provide both deep mechanistic insights and practical engineering guidelines to accelerate the development of next-generation DRX-based lithium-ion batteries.

## 2 Lithium-ion transport in DRX

### 2.1 Li^+^ percolation network

Early studies have suggested that rocksalt-structured oxides with the *Fm*3̄*m* space group generally exhibit poor electrochemical activity, with LiFeO_2_ as a representative.^20^ Owing to their close-packed oxygen lattice and the absence of continuous long-range Li^+^ diffusion pathways, these materials were long considered unsuitable as cathodes and thus received limited attention. In conventional layered cathodes (*R*3̄*m*), oxygen anions form a face-centered cubic (FCC) lattice, while Li^+^ and TM cations occupy octahedral sites arranged in alternating (111) planes. Li^+^ migration in such layered structures proceeds between adjacent octahedral sites *via* an intermediate tetrahedral site, following an octahedral–tetrahedral–octahedral (o–t–o) hopping mechanism.^21^ In ordered layered oxides, only 1-TM (one TM and three Li^+^ face-sharing with the tetrahedral site) and 3-TM (three TM and one Li^+^ face-sharing with the tetrahedral site) environments are present. The 1-TM channels constitute a two-dimensional (2D) percolation network. With a typical slab distance of 2.6–2.7 Å, the Li^+^ migration barrier in layered structure is below 500 meV. Consequently, layered materials generally deliver rapid ionic diffusion kinetics.^7^

**Fig. 2:**
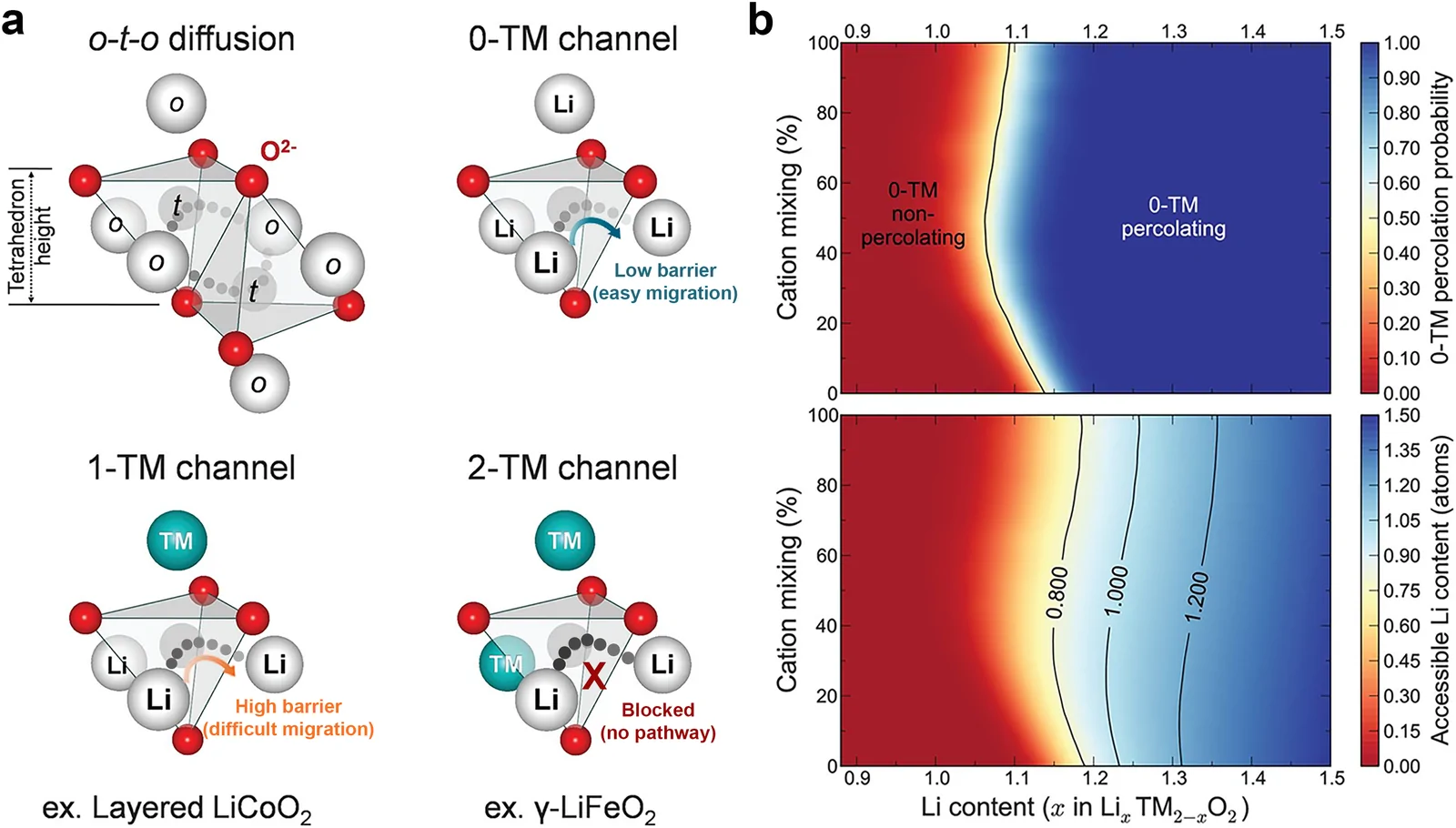
0-TM percolation in DRX materials. (a) Possible environments for an o–t–o Li hop in rocksalt-like Li-TM oxides. (b) Li excess opens a percolating network of 0-TM channels in rocksalt-type Li-TM oxides. Reproduced with permission from ref. 24. Copyright 2014 AAAS.

More recent studies have demonstrated that, at the short-range scale, Li^+^ transport in DRX materials follows a similar o–t–o hopping mechanism.^22,23^ In DRX structures, Li and TM cations randomly occupy a cubic array of octahedral sites, and Li^+^ migrates between neighboring octahedral sites through intermediate tetrahedral positions (Fig. 2a). The occupation of the tetrahedral sites corresponds to an activated state, and the energy of this state largely determines the migration barrier for Li^+^ transport. Accordingly, the factors influencing the Li^+^ migration barrier mainly include: (1) the number and oxidation states of cations occupying the face-sharing octahedral sites, and (2) the size (or height) of the tetrahedral diffusion channel.^24^ Based on the number of TM cations adjacent to the intermediate tetrahedral site, diffusion pathways can be categorized as 0-TM, 1-TM, and 2-TM channels. In general, tetrahedral sites surrounded by two TM cations impose strong electrostatic repulsion on the activated Li^+^, resulting in prohibitively high migration barriers. Consequently, 2-TM channels are largely inactive for Li^+^ transport, whereas 0-TM and 1-TM channels are considered accessible for Li^+^ diffusion.^21^

To compare 0-TM and 1-TM diffusion channels, Ceder *et al.*^24^ employed density functional theory (DFT) to calculate Li^+^ migration barriers in Li_1.211_Mo_0.467_Cr_0.3_O_2_ (LMCO). The results showed that Li^+^ migration through 1-TM channels is associated with high activation energies exceeding 500 meV, whereas migration barriers along 0-TM channels remain below 300 meV. These results indicate that long-range Li^+^ diffusion in DRX is primarily enabled by percolating networks of 0-TM channels. To further elucidate the conditions required for efficient Li^+^ transport in DRX cathodes, a relationship between lithium excess, cation disorder, and the formation of percolating 0-TM diffusion networks was established. Monte Carlo simulations performed on the Li_*x*_TM_2−*x*_O_2_ system revealed that both lithium content and the degree of cation mixing strongly influence the probability of forming continuous 0-TM pathways (Fig. 2b). A minimum lithium content of approximately *x* ∼1.09 is required to form a continuous diffusion network, whereas when the lithium content exceeds *x* ∼1.22, nearly 1 mol of Li^+^ participates in the percolating network regardless of the degree of cation disorder. Experimental observations of high-capacity DRX cathodes are consistent with these theoretical predictions, and a Li excess of approximately 10–20% is typically employed in DRX materials.^25,26^

Although “Li-excess” (in Li_*x*_TM_2−*x*_O_2_, *x* > 1.1) is widely considered a prerequisite for achieving high electrochemical performance in DRX cathodes, recent studies indicate that it is not critical for low voltage TM-based DRX (*e.g.* Mn^3+^, Mo^3+^, V^3+^) nanoparticles.^25^ Specifically, both Li_1.05_Mn_0.90_Nb_0.05_O_2_ and Li_1.20_Mn_0.60_Nb_0.20_O_2_ exhibit high reversible capacities exceeding 250 mAh g^−1^, despite their significantly different lithium contents. In contrast, Li excess remains essential for DRX cathodes incorporating high-voltage TMs (*e.g.*, Fe^3+^, Co^3+^, Ni^2+^, Ni^3+^), independent of particle size. In these systems, high capacities rely predominantly on anionic redox, which requires sufficiently high Li content to stabilize oxidized oxygen species. By comparison, low-voltage TM-based DRXs primarily utilize TM redox reactions and therefore do not require a high degree of Li excess once the particle size is sufficiently reduced.

Furthermore, kinetic Monte Carlo simulations and DFT calculations have revealed that the Li site energy distribution (energy landscape) is the dominant factor governing Li^+^ transport in DRX cathodes.^23,27^ Conventional analyses of ion transport typically assume identical occupancy probability for all Li sites and merely concentrate on diffusion pathways with minimum migration barriers. Nevertheless, the actual Li^+^ transport process in DRXs cannot be comprehensively interpreted only by the 0-TM percolation network, and the influence of site energy heterogeneity must be considered.^27^ Owing to the cation disordered arrangement, DRX structures contain a wide range of Li migration pathways with activation barriers from 0.2 eV to 1.3 eV. The substantial variation in local TM environments leads to significant fluctuations in Li-site energies, where some sites become exceptionally stable and form deep traps. Even with continuous and fully interconnected 0-TM diffusion pathways, a large number of Li^+^ remain trapped in these stable sites and barely participate in diffusion; only a small fraction of Li^+^ located at high-energy sites are mobile. Accordingly, the room-temperature Li^+^ diffusion coefficient is reduced by more than two orders of magnitude relative to theoretical predictions, a phenomenon that cannot be explained by conventional percolation theory. The disordered distribution of TM in DRX cathodes creates a highly heterogeneous Li-site energy landscape, which severely suppresses ionic transport. Hence, understanding Li^+^ diffusion in DRX materials requires simultaneous consideration of two critical factors: 0-TM pathway connectivity and Li-site energy distribution.

### 2.2 Role of short-range ordering (SRO)

**Fig. 3:**
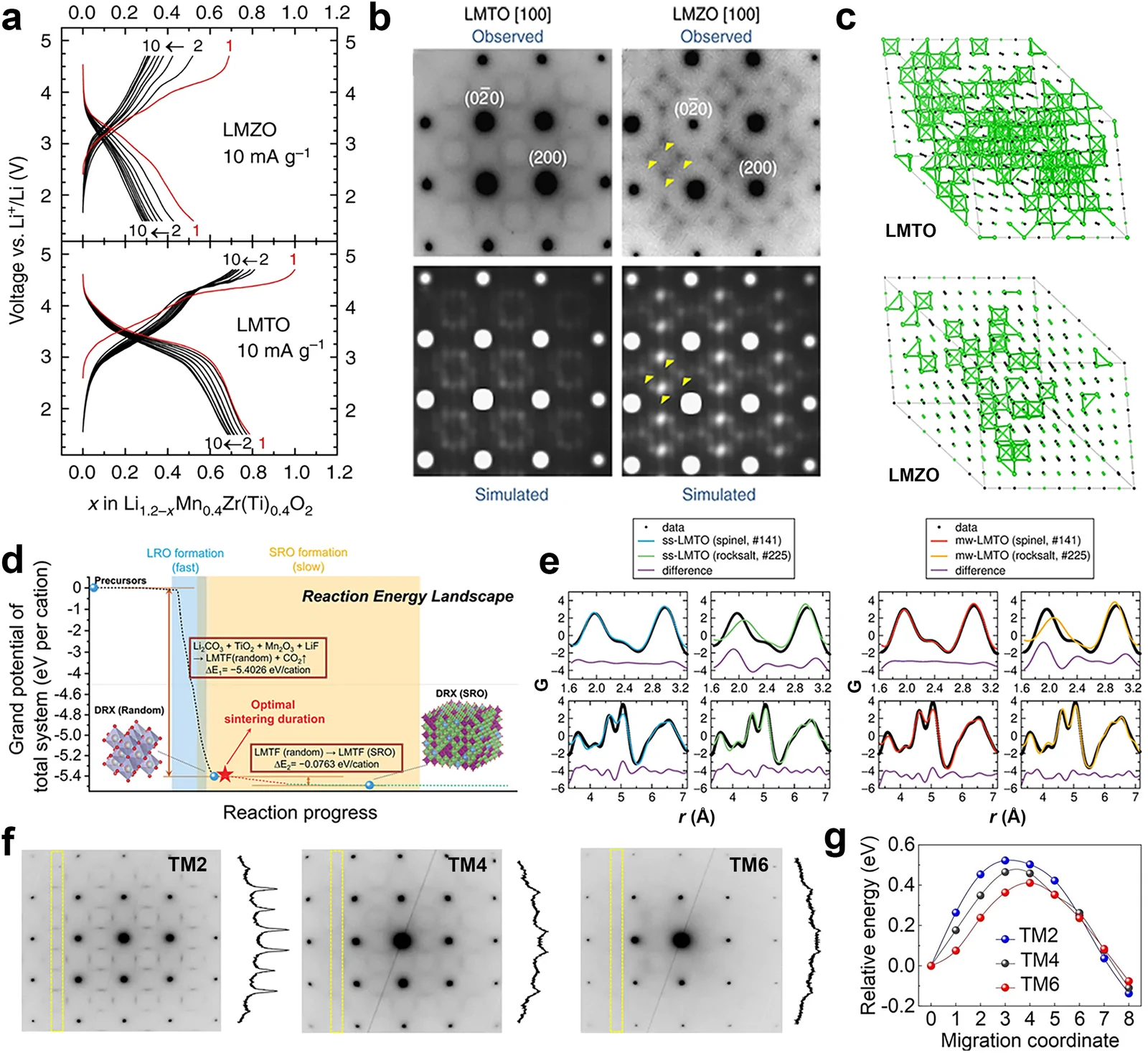
Effect of cation SRO on Li^+^ transport in DRX cathodes. (a) Voltage profiles of LMZO and LMTO. (b) Experimental and simulated electron diffraction patterns for LMTO and LMZO. (c) Representative MC structures for LMTO and LMZO. Reproduced with permission from ref. 28. Copyright 2019 Springer Nature. (d) Schematic of the reaction energy in the LMTF synthesis process. Reproduced with permission from ref. 33. Copyright 2022 Wiley-VCH. (e) X-ray PDF fitting results for ss-LMTO and 5 min microwave-synthesized mw-LMTO. Reproduced with permission from ref. 35. Copyright 2023 Wiley-VCH. (f) TEM selected-area electron diffraction (SAED) patterns for as-synthesized TM2, TM4 and TM6. Reproduced with permission from ref. 36. Copyright 2021 Springer Nature. (g) Li^+^ diffusion barriers along the o–t–o migration path for TM2, TM4 and TM6. Reproduced with permission from ref. 37. Copyright 2023 Wiley-VCH.

If the cation distribution were truly random, DRX oxides with identical Li/TM ratios would exhibit similar populations of Li-migration channels and comparable Li^+^ transport. However, Ji *et al.*^28^ demonstrated that cation SRO plays a critical role in regulating Li^+^ transport in DRX cathodes, as local cation arrangements deviate significantly from ideal randomness at short length scales. Because Li diffusion relies on percolation through a three-dimensional network of interconnected low-barrier migration channels, even subtle variations in local cation configurations can markedly affect the fraction of kinetically accessible Li^+^.^29–31^ This is illustrated by comparing Li_1.2_Mn_0.4_Ti_0.4_O_2_ (LMTO) and Li_1.2_Mn_0.4_Zr_0.4_O_2_ (LMZO). Despite the larger ionic radius of Zr^4+^, which is expected to expand the lattice and promote Li^+^ migration, LMZO exhibits inferior electrochemical performance compared to LMTO (Fig. 3a). This contrast arises from differences in cation SRO. Although both materials share a disordered rocksalt structure at long-range scale, electron diffraction reveals distinct SRO features, with LMZO exhibiting stronger diffuse scattering, indicative of more pronounced SRO (Fig. 3b). Furthermore, the configurations differ fundamentally: LMTO is dominated by octahedral cation clusters, whereas LMZO favors tetrahedral cation clusters. Neutron pair distribution function (NPDF) refinement further confirms that a random model adequately describes LMTO but fails for LMZO, indicating a higher degree of local ordering in LMZO. These differences in SRO strongly influence Li percolation behavior. Consequently, LMTO forms well-connected 0-TM diffusion networks that enable efficient Li^+^ transport, whereas the pronounced SRO in LMZO disrupts the connectivity of 0-TM channels, leading to sluggish Li^+^ diffusion (Fig. 3c).

Collectively, Li^+^ transport in DRX cathodes is governed by the interplay among 0-TM percolation, SRO, and the heterogeneous Li-site energy landscape. A continuous 0-TM percolation network serves as the prerequisite for long-range Li^+^ migration; without sufficient Li-excess or cation disorder, percolation remains the main kinetic limitation. Upon crossing the 0-TM percolation threshold, unfavorable SRO emerges as the primary bottleneck. However, even when percolation is established and SRO is mitigated, the heterogeneous Li-site energy landscape can still trap Li^+^ and hinder transport. Importantly, SRO directly influences the Li-site energy distribution, where optimized SRO narrows the energy spread to promote ionic conduction.^23^ Because this broad energy landscape inherently stems from the disordered arrangement of cations with varied valence states and ionic radii,^27^ tuning the Li-site energy distribution is indispensable for achieving rapid Li^+^ transport kinetics in Li-excess DRX cathodes.

### 2.3 Strategies for enhancing Li^+^ transport

#### 2.3.1 Suppressing unfavorable SRO

To minimize the adverse effects of unfavorable SRO on the Li^+^ percolation network, various synthetic strategies have been developed to suppress its formation. Specifically, DRX materials with identical compositions but different degrees of SRO can be obtained by tuning the high-temperature sintering time.^32^*In situ* characterization combined with *ab initio* thermodynamic grand potential calculations demonstrate that the long-range disordered rocksalt framework forms rapidly, whereas SRO develops over a much longer time scale (Fig. 3d). For example, Li_1.2_Mn_0.55_Ti_0.25_O_1.85_F_0.15_ (LMTF) sintered at 1000 °C for 35 min exhibits significantly less SRO than that sintered for 4 h, resulting in improved electrochemical performance.^33^ Consequently, short-duration high-temperature synthesis generally yields DRX materials with high specific capacity and excellent rate capability, delivering (313 mAh g^−1^ at 20 mA g^−1^ and 140 mAh g^−1^ at 2000 mA g^−1^), both of which are significantly higher than those of the sample synthesized for 4 h (LMTF). Building on this concept, Park *et al.*^34^ developed a rapid and energy-efficient Joule-heating strategy to synthesize high-performance Mn-DRX cathodes. The Joule-heating-synthesized LMTO (1000 °C, 10 min) exhibits reduced SRO compared to furnace-synthesized counterpart, delivering improved rate performance (161 *vs.* 137 mAh g^−1^ at 1 A g^−1^). However, excessively short processing results in insufficient microstructural development, whereas prolonged treatment promotes excessive SRO formation; both are detrimental to electrochemical performance. In addition, Clément *et al.*^35^ reported a microwave-assisted synthesis technique that curtails reaction times to just 5 min while affording precise control over particle size and morphology. Despite the substantially accelerated reaction and quenching rates compared to conventional solid-state pathways, diffuse electron scattering associated with SRO is still observed, and PDF refinements further indicate deviations from the random cation distribution model at short correlation lengths (Fig. 3e). This is likely driven by the elevated temperatures achieved during microwave processing, which accelerate SRO formation kinetics. Collectively, these studies demonstrate that while rapid synthesis can effectively suppress short-range ordering, stringent control over both temperature and processing duration remains essential to mitigate excessive SRO and ensure superior electrochemical performance.

Introducing configurational entropy into DRX has emerged as an effective strategy for suppressing unfavorable SRO. In DRX systems, when the total metal content is fixed, increasing the diversity of transition-metal cations (TMs) reduces the degree of SRO while enhancing energy density and rate capability. As shown in Fig. 3f, DRX cathodes containing 2, 4, and 6 types of TMs were compared, and the degree of SRO was evaluated using transmission electron diffraction. The intensity of diffuse scattering associated with SRO progressively decreases with increasing TM diversity, indicating that increasing the number of TM species from 2 to 6 effectively suppresses SRO in the DRX structure. To further advance the design of high-entropy DRX materials, a compatibility analysis of 23 TMs was conducted, and single-phase high-entropy DRX compounds containing up to 12 TM species were successfully synthesized, demonstrating the feasibility of this design strategy.^36^ However, the capacity retention of high-entropy Mn-based DRX is relatively poor, which may be mainly ascribed to the disproportionation of Mn^3+^ accompanied by the dissolution of Mn^2+^. Therefore, a new class of Ni^2+^–Nb^5+^-based high-entropy DRXs was developed to systematically explore the structural and chemical evolution of DRX electrodes with an increasing number of TM components during cycling.^37^ Benefiting from the high-entropy effect, the optimized TM6 delivers an initial discharge capacity of 277.6 mAh g^−1^ at 20 mA g^−1^ and retains 62.4% of its capacity at 1000 mA g^−1^, which stems from suppressed local cation ordering, a more randomized cation distribution, and abundant continuous 0-TM diffusion channels. *In situ* EIS measurements show that TM6 exhibits a lower charge-transfer resistance (*R*_ct_) than TM2, indicating faster Li^+^ diffusion, which corroborates TM6's lower SRO and better rate capability compared to TM2. DFT calculations (Fig. 3g) further confirm that the high-entropy electrode possesses a significantly lower Li^+^ diffusion barrier.

Although the high-entropy strategy can enhance Li^+^ diffusion kinetics by suppressing unfavorable SRO, increasing the number of cation species does not necessarily lead to improved performance. Squires and Scanlon demonstrated that while increasing cation diversity suppresses SRO and expands the 0-TM Li^+^ percolation network, these structural benefits exhibit diminishing returns as additional transition metals are introduced.^38^ Critically, SRO is fundamentally driven by inherent TM–O and Li–F bonding preferences and therefore cannot be fully eliminated by configurational entropy alone. Consequently, the rational design of high-entropy DRX cathodes requires the synergistic optimization of TM composition, Li excess, and F content, rather than simply maximizing the number of elemental components. Fluorination also represents an effective anion-engineering strategy for DRX cathodes, which enhances structural stability, suppresses irreversible oxygen loss, and regulates SRO.^39,40^ By modulating the local coordination environment and promoting Li^+^ percolation pathways, fluorination improves Li^+^ diffusion kinetics and rate capability. Meanwhile, it stabilizes TMs redox and mitigates interfacial side reactions, thus boosting reversible capacity and cycling stability. Notably, Lun *et al.*^41^ revealed that structural fluorination promotes the formation of Li_4_ tetrahedral (0-TM) environments by electrostatically attracting Li^+^, thereby increasing the density of highly mobile 0-TM units. Excessive fluorination, however, induces localized lithium segregation into isolated clusters, disrupting the continuity of long-range percolation pathways and lowering overall Li^+^ connectivity. Consequently, while optimized fluorination strategically tailors the local coordination environment to enhance electrochemical performance, over-doping imposes severe structural constraints that impede macroscopic Li^+^ migration.

#### 2.3.2 Formation of the δ phase in Mn-rich DRX

**Fig. 4:**
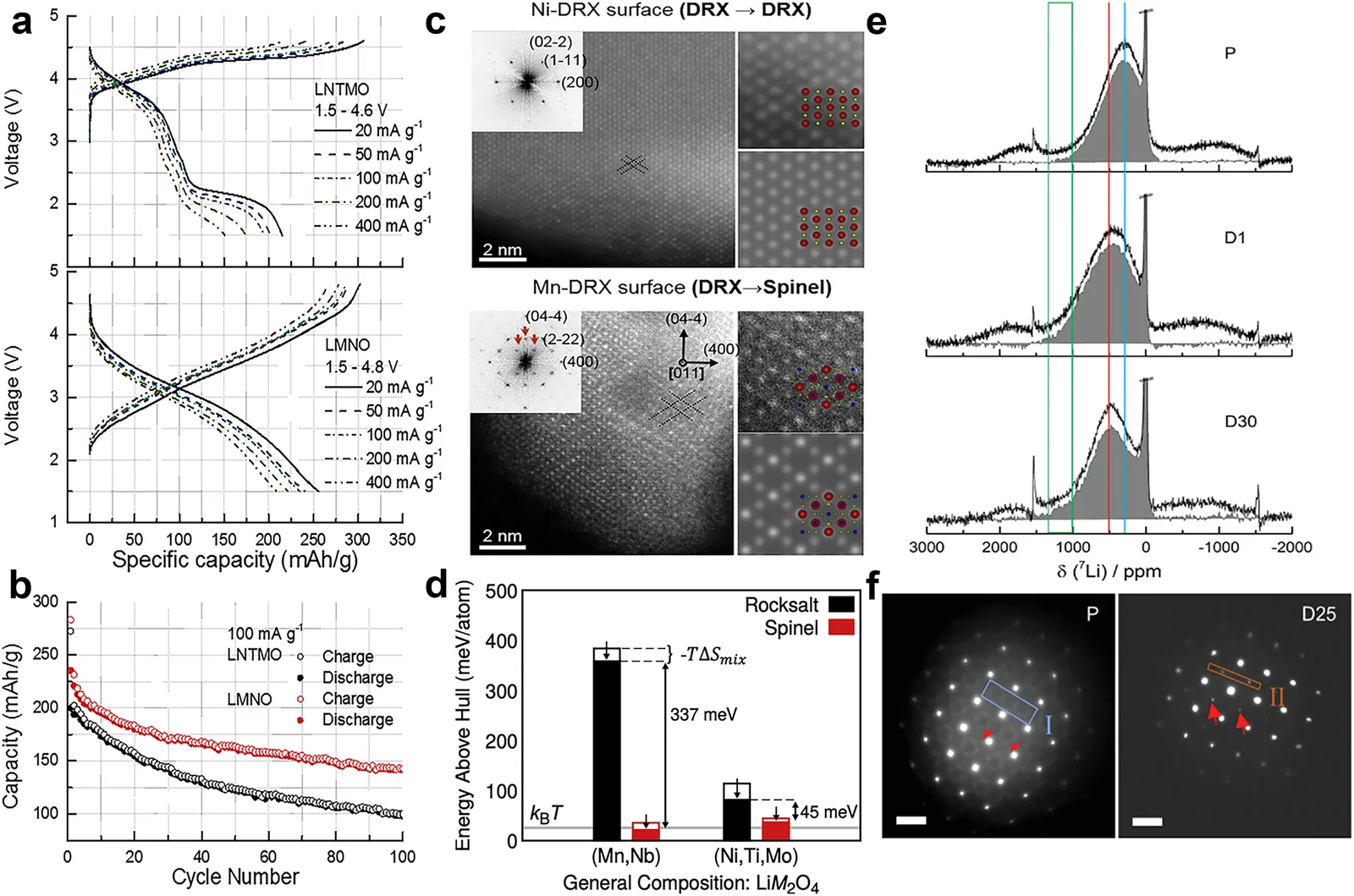
Effect of surface structural evolution on the electrochemical performance of DRX cathodes. (a) Rate performance of LNTMO and LMNO. (b) Capacity retention of LNTMO (black) and LMNO (red) during cycling. (c) Atomic-resolution STEM images of the surfaces of cycled LNTMO and LMNO. (d) Relative formation energies of rocksalt and spinel structures for LMNO and LNTMO. Reproduced with permission from ref. 43. Copyright 2020 Elsevier. (e) ^7^Li spin echo MAS NMR spectra (black lines) and pj-MATPASS isotropic spectra (grey shaded areas). (f) SAED patterns of the pristine cathode (P) and the electrode after the 25th discharge process (D25) viewed along [001] zone axis. Reproduced with permission from ref. 44. Copyright 2022 Wiley-VCH.

Benefiting from the low cost and natural abundance of Mn, Mn-rich disordered rocksalt cathodes are considered promising candidates for low-cost, sustainable, high-energy-density storage. Recent studies have demonstrated that Mn-rich DRX undergoes structural and electrochemical evolution during cycling, leading to improved electrochemical behavior.^42^ Such evolution preferentially occurs in compositions characterized by a high Mn content and low d^0^ cation concentration, particularly when the manganese fraction exceeds ∼0.6 (*e.g.* Li_1.1_Mn_0.7_Ti_0.2_O_2_). During cycling, these materials gradually evolve into a spinel-like δ phase characterized by partial cation ordering and a short coherence length. This unique structure maintains a highly accessible Li^+^ percolation network, thereby enhancing Li^+^ transport kinetics. Ceder *et al.*^43^ first elucidated the impact of surface structural transformations on electrochemical properties by comparing two distinct DRX formulations, Li_1.2_Ni_0.333_Ti_0.333_Mo_0.133_O_2_ (LNTMO) and Li_1.2_Mn_0.6_Nb_0.2_O_2_ (LMNO). Although both cathodes deliver high initial capacities, LNTMO suffers from severe polarization during extended cycling, whereas LMNO exhibits significantly suppressed polarization. This distinction in electrochemical kinetics is directly reflected in their respective rate capabilities; as the current density increases from 20 to 400 mA g^−1^, the discharge capacity of LNTMO drops by ∼30% (from 215 to 150 mAh g^−1^), whereas LMNO experiences a minor ∼20% decrease (from 255 to 208 mAh g^−1^). Meanwhile, LMNO exhibits superior capacity retention compared with LNTMO (Fig. 4a and b). The origin of these performance differences lies in their distinct surface phase evolution. Specifically, a crystalline spinel phase spontaneously forms on the surface of cycled LMNO, enabling fast Li^+^ transport. In contrast, a cation-densified yet disordered DRX layer remains on the surface of LNTMO, which impedes Li^+^ diffusion (Fig. 4c). DFT calculations further reveal that the spinel phase is more stable than the rocksalt phase in LMNO, providing a strong thermodynamic driving force for surface spinel formation. Conversely, the rocksalt phase in LNTMO is stabilized by its larger variety of TM species and higher mixing entropy, resulting in an extremely weak thermodynamic driving force for spinel formation (Fig. 4d). This result highlights that the formed spinel layer acts as an ideal surface passivation layer, which not only facilitates fast Li^+^ transport but also maintains efficient Li percolation even under low Li-excess conditions. In addition, an interesting phenomenon has been observed in high-energy Mn- and F-rich DRX oxyfluoride cathodes: a capacity increase over 125% can be achieved upon cycling.^44,45^ Rather than originating from enhanced oxygen redox activity, the improved capacity and kinetics are attributed to local structural rearrangements within the DRX framework. Comprehensive characterizations have confirmed that off-stoichiometry spinel-like nanodomains form during cycling, while the long-range cubic rocksalt framework remains unchanged. Solid-state ^7^Li NMR reveals the evolution of local Li environments upon cycling, verifying the formation of partially ordered spinel-like domains in which Li^+^ occupy tetrahedral sites (Fig. 4e). The broadened chemical shift distribution indicates increased structural heterogeneity. Furthermore, TEM selected-area electron diffraction (SAED) analyses further verify structural rearrangements. The pristine DRX exhibits a typical disordered rocksalt pattern with diffuse scattering from SRO. After cycling, the SRO disappears, accompanied by the emergence of new diffraction features corresponding to a spinel-like structure (Fig. 4f). Notably, such local structural transformation is commonly observed in various Mn-based DRX cathodes. The interconnected spinel-like domains construct highly efficient 3D Li^+^ diffusion pathways, suppress detrimental structural distortions, and promote the utilization of active material, thereby delivering exceptional cycling stability and progressive capacity improvement.

**Fig. 5:**
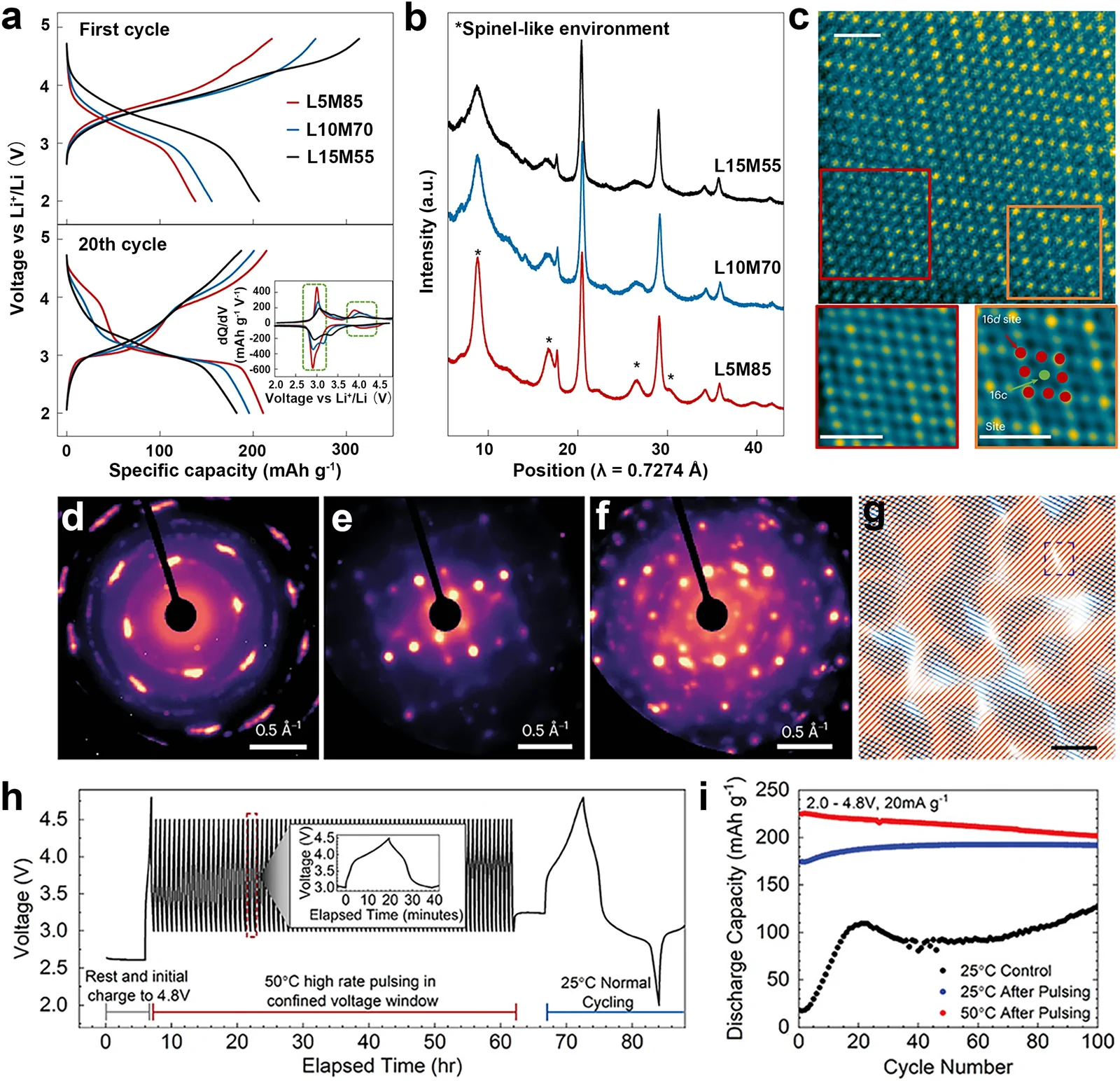
Spinel-like δ-phase formation in Mn-rich disordered rocksalt cathodes. (a) Voltage profiles of L5M85, L10M70 and L15M55 for the first and the 20th cycles when cycled between 2–4.8 V at 20 mAh g^−1^. (b) Synchrotron XRD patterns and (c) HAADF-STEM images of *ex situ* samples discharged to 3.5 V during the 20th cycle and magnified region (scale bar = 1 nm). Reproduced with permission from ref. 46. Copyright 2024 Springer Nature. (d–f) Mean SEND pattern of the pristine L12M65, L07M65-D, and L07M65-DH particles, respectively. (g) Filtered HAADF-STEM image revealing cation ordering in the δ-phase, with spinel lattice fringes separated by antiphase boundaries. Reproduced with permission from ref. 47. Copyright 2024 Springer Nature. (h) Representative electrochemical pulsing activation process, including initial charging to 4.8 V, high-rate pulsing within a confined voltage window at 50 °C, and subsequent cycling at 25 °C. (i) Extended cycling performance of the pulsed material synthesized by the molten-salt method at 25 and 50 °C, together with a control cell without prior pulsing. Reproduced with permission from ref. 50. Copyright 2025 Wiley-VCH.

Collectively, the above studies demonstrate that the formation of spinel-like ordered domains during electrochemical cycling represents an effective strategy to boost Li^+^ transport in DRX cathodes. Such favorable ordering is essentially identified as the δ-phase with a distinct spinel-derived structure, which has been recognized as the key to unlocking fast Li^+^ diffusion in DRX materials. Cai *et al.*^46^ synthesized a series of Mn-DRX materials with varying Li excess and Mn content, including Li_1.05_Mn_0.85_Ti_0.1_O_2_ (L5M85), Li_1.10_Mn_0.7_Ti_0.2_O_2_ (L10M70) and Li_1.15_Mn_0.55_Ti_0.3_O_2_ (L15M55). As shown in Fig. 5a, L15M55 with higher Li excess exhibits a better 0-TM percolation network and delivers a higher initial discharge capacity. Notably, after 20 cycles, pronounced plateau-like features appear in L5M85 and L10M70. The migration and rearrangement of Mn during the transformation toward a spinel-like ordering lead to significant changes in the voltage profiles, including increased capacity and the emergence of distinct plateaus at approximately 3 and 4 V *versus* Li/Li^+^. *Ex situ* synchrotron XRD patterns (Fig. 5b) reveal that, besides the main rocksalt reflections, L5M85 develops broad extra peaks after cycling, consistent with δ-phase formation. High-angle annular dark-field scanning transmission electron microscopy (HAADF-STEM) images (Fig. 5c) further confirm the presence of spinel-like local domains, where Mn/TM cations partially occupy 16d and 16c sites with a very short coherence length (∼5 nm). Despite featuring spinel-like ordering, the transformed phase differs from fully ordered spinel in its voltage profile, exhibiting more sloped features in the approximately 3 and 4 V regions. Accordingly, this phase is defined as the δ phase, which enables the material to avoid the two-phase reaction of well-ordered spinel while retaining the favorable rate capability of spinel-type structures.

Although the *in situ* formation of the δ-phase during electrochemical cycling provides an effective route to regulate local structure and Li storage behavior in Mn-rich DRX cathodes, the transformation process is extremely slow (typically up to three weeks), resulting in poor initial electrochemical performance. Recent studies have demonstrated that chemical delithiation followed by low-temperature annealing can induce the formation of partially ordered spinel domains (3–7 nm) in Mn-rich DRX materials.^47^ To further elucidate the structural evolution during this process, four-dimensional scanning electron nanodiffraction (4D-SEND) was employed. As shown in Fig. 5d, the pristine DRX particle exhibits sharp diffraction spots, indicating a highly crystalline structure. After chemical delithiation, the mean SEND pattern of the Li_0.7_Mn_0.65_Ti_0.15_O_1.9_F_0.1_ (L07M65-D) particle (Fig. 5e) reveals that the δ-phase preferentially forms within the near-surface region (∼50 nm), suggesting that the DRX-to-δ phase transformation initiates from the particle surface during delithiation. Following heat treatment, the entire particle uniformly transforms into the δ-phase with significantly enhanced diffraction intensity (Fig. 5f), highlighting the critical role of thermal treatment in promoting complete phase conversion. In addition, abundant antiphase boundaries between differently oriented spinel domains are observed (Fig. 5g), together with thin disordered regions separating adjacent spinel-like domains. The superior electrochemical performance of the δ-phase is mainly attributed to its nanoscale spinel-like domains with short coherence lengths. Such structural features effectively suppress detrimental two-phase reactions by transforming first-order phase transitions into continuous solid-solution behavior, thereby eliminating the characteristic 3 V voltage plateau and alleviating the associated phase-transition strain. As a result, the L07M65-DH cathode achieves an initial discharge capacity of 201 mAh g^−1^ despite a large particle size (5 µm), significantly higher than the 159 mAh g^−1^ of Li_1.2_Mn_0.65_Ti_0.15_O_1.9_F_0.1_ (L12M65). In contrast, conventional DRX materials typically require nanosizing (<500 nm) to achieve high capacity. For example, Li_1.3_Mn_0.4_Nb_0.3_O_2_ delivers only 101 mAh g^−1^ at the micrometer scale, while the Li_1.05_Mn_0.85_Ti_0.1_O_2_ shows only 62 mAh g^−1^ without particle size reduction.^48,49^ These results demonstrate that δ-phase engineering enables high capacity in micrometer-sized particles, breaking the conventional dependence of DRX cathodes on nanosizing and offering a promising strategy for scalable Mn-based cathodes. However, this approach involves complex processing, strict temperature control, and limited scalability, which hinder practical application. Li *et al.*^8^ further clarified that δ-phase transformation occurs only in Mn-rich DRX materials and is governed by Mn content. By comparing Mn-rich Li_1.1_Mn_0.7_Ti_0.2_O_2_ (LMT72) and Mn-poor Li_1.2_Mn_0.4_Ti_0.4_O_2_ (LMT44) after chemical delithiation and heating, they showed that Mn-rich DRX exhibits a large thermodynamic driving force (Δ*H* = −19.4 kJ mol^−1^) and low phase transition barrier. It forms coherent spinel domains that share the same FCC oxygen framework with the DRX matrix, where Li^+^ occupies tetrahedral 8a sites and Mn/Ti order at 16c/16d sites. In contrast, Mn-poor DRX shows negligible driving force and forms incoherent spinel domains with lattice mismatch and strain, preventing bulk δ-phase formation during cycling. Therefore, Mn-rich compositions (Mn ≥ 0.5) are essential for enabling δ-phase formation, efficient capacity activation, and stable cycling in DRX cathodes.

Recently, an alternative approach has been developed that leverages electrochemical pulsing to rapidly convert Mn-rich DRX materials into the structurally optimized δ-phase.^50^ This method can shorten the transformation time of DRX to the spinel-like δ-phase to within one day. Such rapid conversion is achieved through repeated charge–discharge cycling within a confined voltage window of 3.0–4.5 V at elevated temperature and high rate. The complete voltage–time profile of the pulsing activation process is shown in Fig. 5h. Furthermore, this method can also activate micron-sized single-crystal DRX particles without the need for shake-milling with carbon. Li_1.1_Mn_0.8_Ti_0.1_O_1.9_F_0.1_ single crystals with particle sizes of 2–3 µm were synthesized *via* a molten-salt method and successfully transformed into the δ-phase after pulsing activation. When cycled at 50 °C, the activated material delivered a discharge capacity of 225 mAh g^−1^ and an energy density of 700 Wh kg^−1^, while its relatively low specific surface area contributed to excellent cycling stability (Fig. 5i). The emergence of the δ-phase in Mn-rich DRX cathodes represents an effective strategy for tuning local structure to enhance electrochemical performance. It exhibits a unique nanoscale architecture with short-range spinel-like ordering within a partially disordered cation framework, enabling fast Li^+^ transport and suppressing spinel-like two-phase reactions, thereby delivering improved rate capability even in micron-sized particles.

Despite these promising attributes, the implementation of the δ-phase strategy entails several critical limitations for practical application. Compositionally, the δ-phase formation is restricted to Mn-rich DRX formulations, which severely constrains the design space while aggravating issues related to Mn dissolution and Jahn–Teller distortion during extended cycling.^37^ From a processing perspective, existing δ-phase activation routes remain labor-intensive and time-consuming, and scalable manufacturing is hampered by the absence of rapid synthesis protocols. Moreover, the cycling stability of the δ-phase has not been sufficiently validated; existing literature predominantly reports stability for roughly 100 cycles between 2 and 4.8 V, which is far below the 500–1000 cycle benchmark required for commercial energy storage.^47,50^ Consequently, future efforts must focus on establishing simpler, faster, and more energy-efficient activation approaches.

#### 2.3.3 Partially disordered compounds

**Fig. 6:**
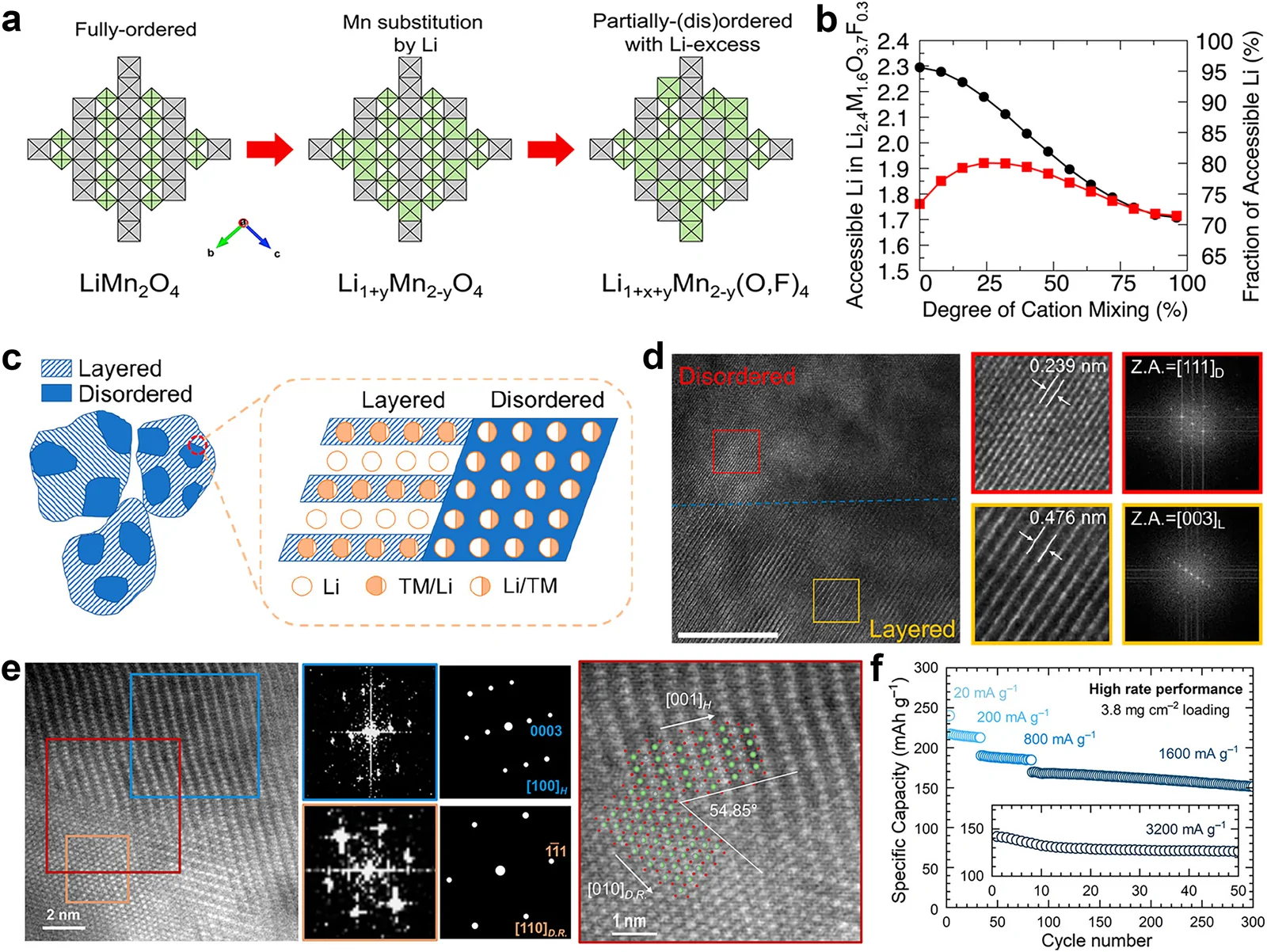
Design and characterization of partially (dis)ordered cathodes. (a) Schematic illustration of the evolution from fully ordered LiMn_2_O_4_ to partially (dis)ordered Li-rich compositions through Li substitution for Mn (gray octahedra = MnO_6_, green tetrahedra = LiO_4_). (b) Accessible Li content and fraction of accessible Li as a function of the degree of cation mixing (disorder). Reproduced with permission from ref. 55. Copyright 2021 Elsevier. (c) Schematic illustration of the layered/disordered coexisting structure in LNMN20. (d) Representative TEM image, local magnifications, and fast Fourier transformation images of LNMN20. Reproduced with permission from ref. 58. Copyright 2024 American Chemical Society. (e) HAADF-STEM image of an interfacial region observed within LiNi_0.925_Ti_0.05625_O_2_ showing co-existence of the ordered *R*3̄*m* (blue) and disordered *Fm*3̄*m* rock salt (orange) domains oriented along [100]_H_ and [110]_DR_, respectively, compared with the simulated patterns (right). (f) High-rate and long-term cycle performance for LiNi_0.925_Ti_0.05625_O_2_. Reproduced with permission from ref. 59. Copyright 2025 Wiley-VCH.

In addition to optimizing compositions and SRO as well as forming spinel-like δ-phase domains, partially ordered structures have been rationally designed as ideal intermediates between fully ordered and fully disordered materials.^51,52^ These systems feature a flatter energy landscape that provides favorable local coordination environments for facile Li^+^ transport, thereby significantly enhancing rate capability. By retaining a certain degree of long-range order while strategically introducing structural disorder, these configurations suppress phase transitions within the parent lattice. This structural synergy results in smoother voltage profiles, mitigated site-energy variations, and vastly improved Li^+^ transport kinetics. Two representative examples illustrating this concept are spinel-like and layered-like ordering. For conventional spinel LiMn_2_O_4_, the introduction of 10–20% cation disorder can convert the two-phase region at 3 V into a solid-solution process, enabling stable cycling over an extended voltage range.^53^ Notably, such partially disordered spinel materials can be synthesized *via* mechanochemical ball milling.^54^ Along this direction, a continuous order–disorder tuning strategy has been developed. By introducing excess Li and fluorine doping into the Li_1.4+*x*_Mn_1.6_O_3.7_F_0.3_ system, a controllable and continuous structural evolution from an ordered spinel to a partially disordered spinel and further to a rocksalt-like phase is achieved (Fig. 6a).^55^ The partially disordered spinel fully suppresses the two-phase reaction at 3 V and enables smooth solid-solution behavior, while retaining the intrinsic three-dimensional fast Li^+^ diffusion pathways of the spinel framework. Electrochemical results show that all three cathode materials deliver a capacity of >350 mAh g^−1^ and an energy density of >1000 Wh kg^−1^ at 50 mA g^−1^ in a wide voltage range between 1.5 and 4.8 V, while Li_1.47_Mn_1.6_O_3.7_O_0.3_ maintains 158 mAh g^−1^ even at a high rate of 7500 mA g^−1^. This behavior arises because spinel-type cation ordering favors the formation of 0-TM Li^+^ diffusion channels, enabling fast kinetics even with partial disorder. As the degree of disorder increases, the population of 0-TM channels and accessible Li capacity decrease, eventually approaching those of layered or DRX structures (Fig. 6b). In another example of the benefits of partial disorder, Ji *et al.*^56^ constructed PDS materials by synergistically introducing excess Li and F into Mn-based oxyfluorides, overcoming the limitations of fully ordered spinel. The representative composition Li_1.68_Mn_1.6_O_3.7_F_0.3_ exhibits controlled cation disorder, with Mn distributed over both 16d and 16c sites. This structure eliminates the two-phase reaction and voltage plateau near 3 V, enabling smooth solid-solution electrochemical behavior. Benefiting from efficient 0-TM fast Li^+^ diffusion channels associated with spinel-like local ordering, the material achieves both ultrahigh energy density (>1100 Wh kg^−1^) and outstanding rate capability with stable discharge even at 20 A g^−1^. This work highlights a general strategy for designing partially ordered materials bridging ordered and disordered structures.

Although lithium transition metal oxides exhibit high-capacity, their structural instability at high operating voltages (>4.3 V) severely deteriorates battery performance. To address this critical challenge, Pei *et al.*^57^ proposed a medium-entropy configuration tuning strategy, through which Li-rich layered oxide Li_1.2_Ni_0.2_Mn_0.6_O_2_ is transformed into a defective spinel-structured Li_1.46_Ni_0.32_Mn_1.2_O_4−*x*_*via* proton exchange and low-temperature calcination. Upon initial delithiation, the material evolves into a medium-entropy spinel phase with partial cation disorder, in which TM ions occupy both 16d and 16c sites, thereby disrupting the ordered arrangement of conventional spinel. Further structural characterization and theoretical calculations confirm the presence of controllable cation disorder and reveal that Li^+^ migrate between octahedral sites within the spinel framework. In addition to spinel-based entropy engineering, Cui and co-workers developed a descriptor correlating ionic radius and electronic configuration to guide the formation of layered, disordered, and intergrown nanostructures.^58^ Such nanoscale coherent intergrowths combine fast Li^+^ diffusion from layered phases with the structural stability of disordered rocksalt phases, thereby overcoming the limitations of phase transitions in layered oxides and sluggish kinetics in disordered systems (Fig. 6c). Guided by this principle, Li_1.2_Ni_0.4_Mn_0.2_Nb_0.2_O_2_ (LNMN20) delivers a high capacity of 312 mAh g^−1^ with excellent rate capability and cycling stability. HRTEM reveals a two-phase intergrown structure in which disordered regions are embedded within a layered matrix. TEM images further confirm the coexistence of layered and disordered domains, as evidenced by distinct atomic arrangements and corresponding fast Fourier transform patterns (Fig. 6d). This nanoscale intergrowth suppresses structural distortion and oxygen loss while enabling reversible cationic and anionic redox, providing a controllable design strategy for high-performance Li-excess cathodes.

While LiNiO_2_ delivers high capacity and favorable Li^+^ mobility, it fundamentally suffers from severe Li/Ni cation mixing and structural instability upon electrochemical cycling. Lim *et al.*^59^ demonstrated that Ti^4+^ substitution in LiNiO_2_ enables new solid solution compositions of LiNi_1−*x*_Ti_3*x*/4_O_2_. For 0.025 ≤ *x* ≤ 0.2, HAADF-STEM reveals nanoscale coexistence of ordered (*R*3̄*m*) and disordered (*Fm*3̄*m*) rocksalt domains (Fig. 6e). These nanocomposites combine fast kinetics with enhanced structural stability, enabling high-capacity retention and excellent rate performance even at high mass loadings (Fig. 6f). As the result, they exhibit ultralow strain and outstanding cycling stability among the best reported for LiNiO_2_-based cathodes. Years of research have indicated that zigzag-type LiMnO_2_ (denoted as zLMO) is less favorable for cathode applications than other Mn-based oxides, as its redox activity is largely “locked” by the zigzag structure. Despite extensive exploration of Mn-based cathode materials, the Li–Nb–Mn^3+^–O system remains relatively underexplored, representing a critical knowledge gap between zigzag-ordered and fully disordered Li–Mn-based oxides. Recently, a partially cation-disordered lithium niobium manganese oxide featuring a zigzag framework was reported, providing critical insights into this structural continuum.^60^ The introduction of a trace amount of Nb induces approximately 28% Li/Mn site mixing, which significantly activates the Mn^3+^/Mn^4+^ redox couple, suppresses detrimental Mn^3+^ disproportionation, and triggers a unique structural evolution pathway. Specifically, the material transitions from a partially disordered zigzag phase to disordered λ-MnO_2_ during charging, followed by a transformation into a tetragonal disordered rocksalt phase upon discharging. Concurrently, manganese dissolution during electrochemical cycling is markedly mitigated compared to both zLMO and spinel LiMn_2_O_4_, successfully enabling the simultaneous realization of high capacity, enhanced cycling stability, and low cost in Mn-based cathodes.

#### 2.3.4 Introducing additional diffusion pathways

**Fig. 7:**
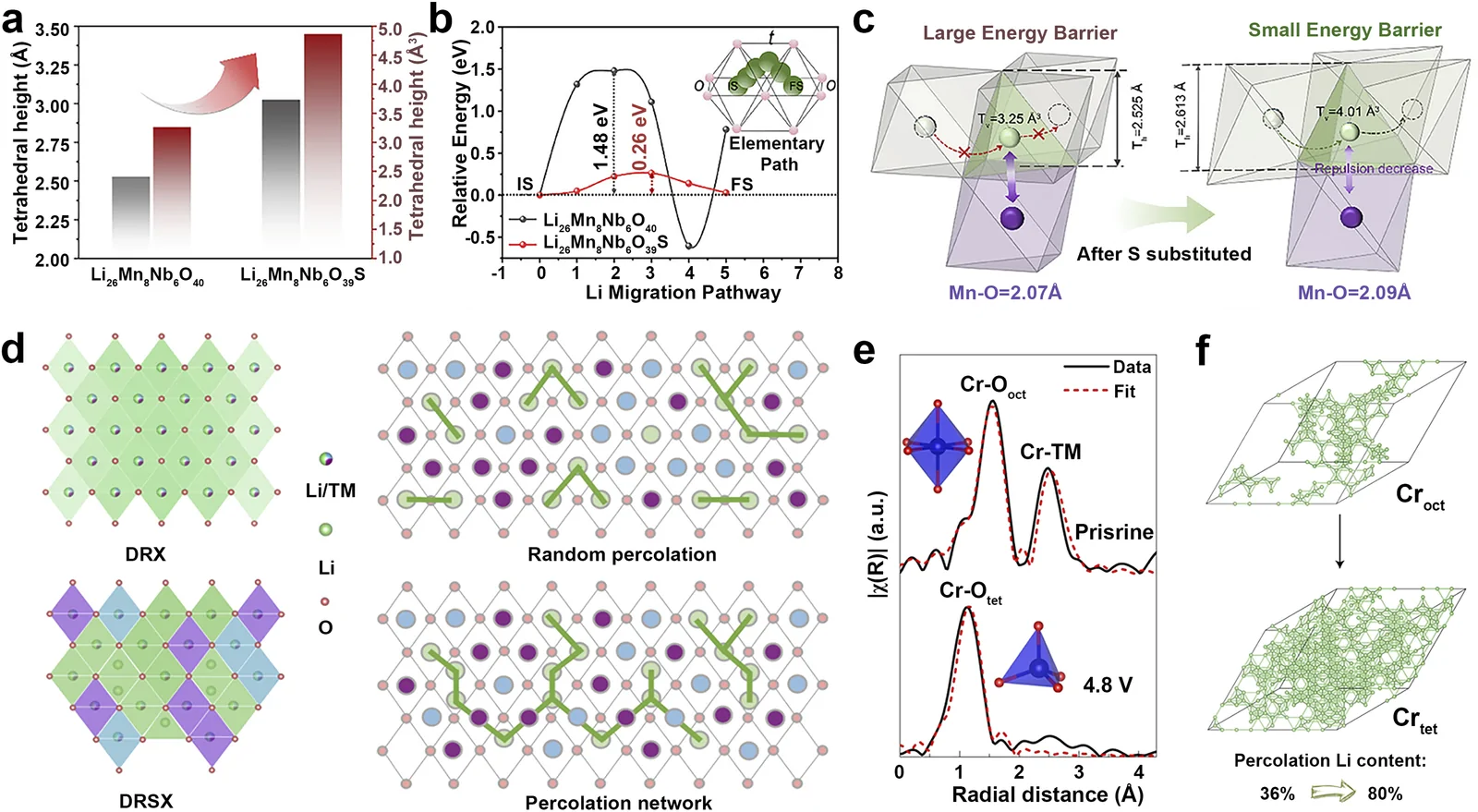
Activating additional diffusion pathways to improve Li^+^ transport kinetics in DRX cathodes. (a) Lattice parameters and volume obtained by Neutron diffraction Rietveld refinement. (b) The energy barriers of 1-TM channel for Li_26_Mn_8_Nb_6_O_40_ and Li_26_Mn_8_Nb_6_O_39_S. (c) Schematic representation of the effect of S-substitution on the reduction of electrostatic repulsion on *t*-Li^+^. Reproduced with permission from ref. 61. Copyright 2026 Wiley-VCH. (d) Illustration of DRX and DRSX structure and illustration of the lithium percolation mechanism. Reproduced with permission from ref. 62. Copyright 2025 American Chemical Society. (e) Fitted Cr K-edge extended X-ray absorption fine structure (EXAFS) spectra of LMTC02O in pristine and 4.8 V-charged states. (f) Representative Monte Carlo-simulated structures for LMTC02O with octahedral Cr and tetrahedral Cr. Reproduced with permission from ref. 65. Copyright 2021 Springer Nature.

In conventional DRX cathodes, long-range Li^+^ diffusion predominantly relies on the percolating 0-TM channels, which typically require a substantial Li excess and are highly sensitive to SRO. To overcome these intrinsic kinetic limitations, Huang *et al.*^61^ proposed an electrostatic-repulsion regulation strategy to activate the otherwise inert 1-TM Li^+^ migration channels, thereby improving Li^+^ transport and electrochemical performance. This approach utilizes the strategic incorporation of chalcogen elements (S or Se), which serve as oxygen congeners with larger ionic radii and lower electronegativity, into the oxide lattice. Systematic structural characterization and computational calculations reveal that in the optimized electrode, the tetrahedral height of 1-TM channels is significantly increased from 2.526 to 2.613 Å, accompanied by a volume expansion from 3.254 to 4.010 Å^3^ (Fig. 7a). Consequently, the Li^+^ migration barrier for 1-TM pathways is drastically reduced from 1.48 to 0.26 eV, below the typical barrier of ∼0.3 eV for highly active 1-TM channels in layered oxides (Fig. 7b). This ultra-low barrier confirms the successful activation of 1-TM channels. As illustrated in Fig. 7c, such anion substitution induces considerable geometrical expansion of 1-TM tetrahedral units, which weakens the electrostatic repulsion exerted on Li^+^ at the tetrahedral transition state. This effect establishes a fully interconnected 3D Li^+^ percolation network by combining 0-TM and activated 1-TM pathways, thereby resolving the intrinsic kinetic bottlenecks of DRX cathodes.

Further advancing the kinetic design of DRX cathodes beyond channel activation, strategic manipulation of Li-site occupancy has emerged as an effective approach to constructing continuous Li^+^ transport networks. Amine *et al.*^62^ developed a tetrahedral Li stuffing strategy that transforms conventional DRX into a disordered rocksalt/disordered spinel intergrowth (DRSX) structure. In the pristine DRX structure, Li and TMs are randomly distributed on 4a sites within a face-centered cubic oxygen framework. In contrast, tetrahedral Li stuffing reconstructs the local coordination environment and generates an interwoven rocksalt/spinel framework, where the spinel-like domains provide fast tetrahedron–octahedron–tetrahedron diffusion channels. Unlike conventional DRX, where long-range Li percolation is limited by the insufficient 0/1-TM channels, the DRSX structure enables efficient Li transport by utilizing tetrahedral Li as low-barrier transport hubs (Fig. 7d). HAADF-STEM confirms the random cation distribution in Li_1.25_Mn_0.45_Ti_0.3_O_1.8_F_0.2_ (LMTOF), whereas Li_1.25−*x*_Mn_0.45_Ti_0.3_O_1.8_F_0.2_ (RSLMTOF) exhibits a homogeneous intergrowth of rocksalt and spinel phases throughout the bulk. The introduced tetrahedron–octahedron–tetrahedron pathways lower Li^+^ migration barrier and enhance network connectivity, thereby accelerating Li^+^ transport kinetics. Notably, this structure achieves effective Li percolation without requiring extreme cation disorder, indicating that fast Li^+^ transport can be realized under moderate disorder. This strategy has also been extended to Mn-rich systems. Overall, tetrahedral Li stuffing modulates local cation coordination and constructs a rocksalt/spinel intergrown framework, effectively overcoming the intrinsic kinetic bottlenecks of conventional DRX cathodes.

Rapid and reversible Li^+^ transport is essential for lithium-ion battery operation and is typically achieved through topotactic reactions that preserve the host structure and maintain continuous diffusion pathways.^63,64^ Accordingly, pursuing perfect topotactic reactions has become a general design principle for high-power cathodes, as such reactions maintain structural integrity and minimize kinetic complexities. In contrast to this conventional view, recent studies have demonstrated that the rate capability of Li-excess DRX can be significantly improved when the topotactic reaction is replaced by a non-topotactic reaction.^65^ For example, in Li_1.2_Mn_0.2_Ti_0.4_Cr_0.2_O_2_, Cr^3+^ undergoes reversible octahedral-to-tetrahedral migration at high states of delithiation, converting blocked 1-TM channels into abundant 0-TM pathways and increasing the percolating Li fraction from 36% to 80% (Fig. 7e and f). As a result, the rate capability is significantly enhanced, with the capacity at 1000 mA g^−1^ increasing from 109 to 155 mAh g^−1^. This strategy has also been extended to Ni-based DRX systems, establishing controlled TM migration as a promising paradigm for designing high-power DRX cathodes.

Several mainstream modification strategies for DRX cathodes have been established, including high-entropy design to suppress unfavorable SRO, Mn-rich compositions to enable *in situ* DRX-to-δ phase transformation, and Cr doping to induce non-topotactic reactions that enhance Li^+^ migration. However, the relative efficacy and mutual compatibility among these strategies remain unclear. To address this knowledge gap, Liu *et al.*^66^ systematically compared the effects of high-entropy design, Cr doping, and δ-phase transformation in DRX materials possessing an identical Li content of 1.15 per formula unit. Their findings reveal that the *in situ* formation of spinel-like δ-phase driven by high Mn content exerts the dominant influence on capacity and cycling-stability improvements. Conversely, Cr or multi-element doping dilutes the Mn concentration and attenuates the driving force for this phase transformation, thereby constraining performance gains. Notably, although high-entropy design effectively suppresses SRO and provides capacity benefits under high Li-excess conditions, its efficacy diminishes at low Li-excess levels.^36,66^ Accordingly, rational DRX cathode design should prioritize a high Mn content (≥0.6 per formula unit) while maintaining high Li-excess within the optimal 1.15 to 1.20 range. This specific compositional range preserves sufficient TM-site availability to accommodate the high Mn fractions necessary for δ-phase formation; exceeding a Li-excess of 1.25 reduces total TM occupancy and limits allowable Mn content due to charge-balance constraints. Consequently, fulfilling the structural prerequisites for δ phase formation should be prioritized in the development of high-performance DRX cathodes.

## 3 Electronic transport in DRX cathodes

### 3.1 Electronic transport mechanism

**Fig. 8:**
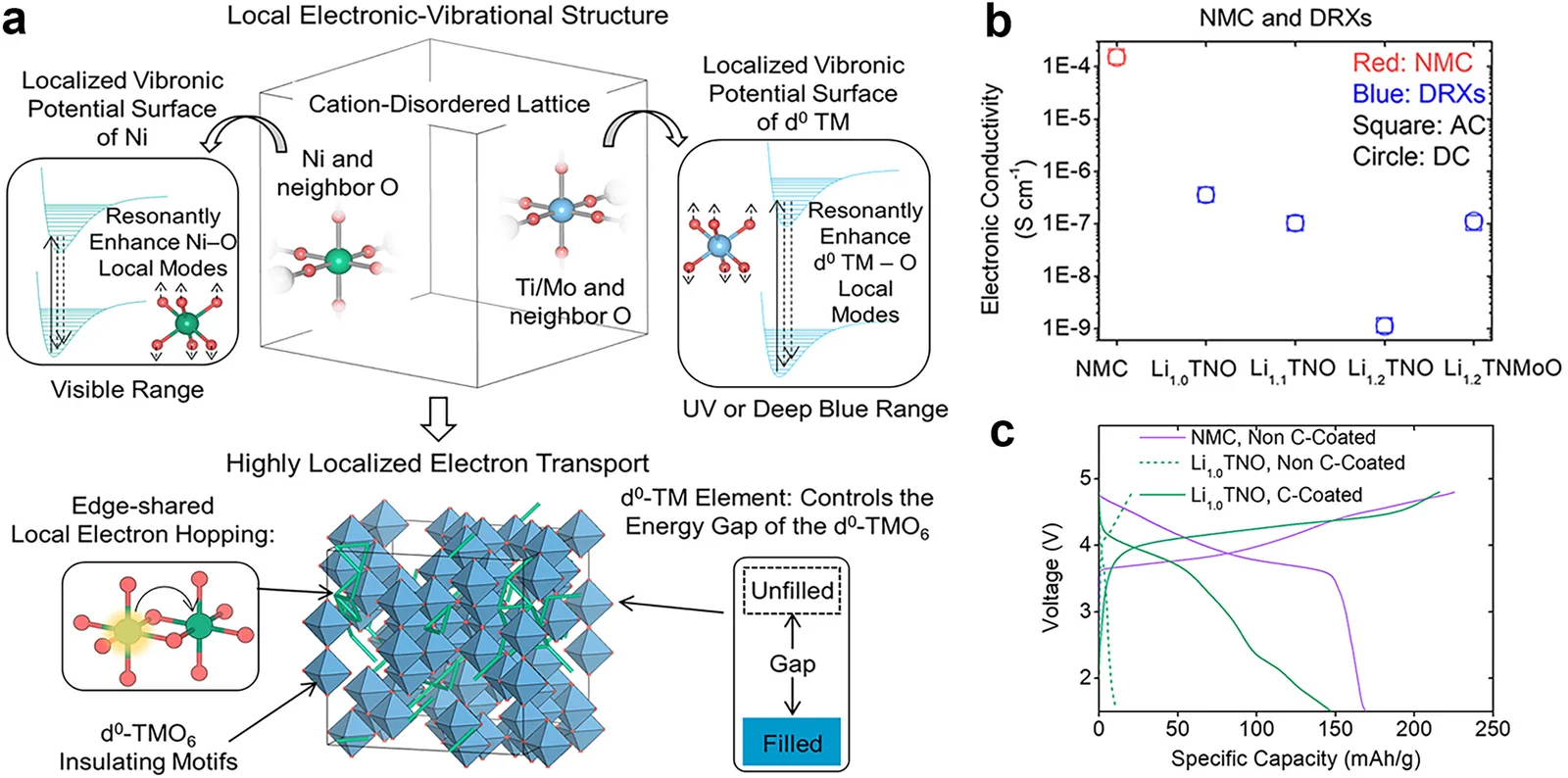
Electronic transport mechanism in DRX cathodes. (a) Schematic illustration of electron localization and the factors governing electron transport in Ni-based DRX cathodes. (b) Electronic conductivity of NMC, Li_*x*_TNO, and Li_1.2_TNMoO powder. (c) Voltage profiles of NMC, non-C-coated, and C-coated Li_1.0_TNO. Reproduced with permission from ref. 18. Copyright 2024 American Chemical Society.

It is well established that the electrochemical kinetics of battery-electrode kinetics rely on both electronic and ionic conduction, rather than Li^+^ transport alone. Notably, in addition to sluggish Li^+^ diffusion, DRX cathodes also suffer from inherently poor electronic conductivity, a critical limitation that has often been overlooked in prior research.^19^ This low conductivity arises from strong electron localization within the disordered cation lattice. Unlike layered oxides with continuous TM conduction pathways, DRX displays a random distribution of redox-active TMs (*e.g.*, Ni, Mn) and insulating d^0^-TMs (*e.g.*, Ti^4+^, Nb^5+^, Mo^6+^) over octahedral sites. With large band gaps and no valence electrons, d^0^-TM octahedra disrupt edge-sharing TM networks and impede long-range electron transport.^67^ Consequently, electron conduction in DRX proceeds primarily *via* small polaron hopping between isolated redox-active TM clusters, yielding conductivities 3–5 orders of magnitude lower than those of layered cathodes, even lower than LiFePO_4_ (∼5 × 10^−8^ S cm^−1^).^68^ Increasing d^0^-TM content further dilutes redox-active sites and aggravates localization, while substituting Ti^4+^ with Mo^6+^ can moderately enhance electron hopping by narrowing the TM–O band gap.^69^ As illustrated in Fig. 8a, DRX exhibits pronounced electron localization. Electron transport occurs through localized hopping between edge-sharing TM octahedra, yet these pathways are fully surrounded and disconnected by insulating d^0^-TM octahedra. Such fragmented electronic architectures, characterized by sparse, isolated conductive paths, represent the fundamental origin of the low conductivity and rate-limiting kinetics in DRX cathodes.

Outka *et al.*^18^ investigated a series of DRX cathodes, including Li_*x*_Ti_*x*/2_Ni_2−3*x*/2_O_2_, (*x* = 1, 1.1, 1.2, denoted as Li_*x*_TNO) and Mo-substituted Li_1.2_Ti_0.333_Ni_0.33_Mo_0.13_O_2_ (Li_1.2_TNMoO), with LiNi_0.33_Mn_0.33_Co_0.33_O_2_ (NMC) as the layered reference. To clarify the role of electronic transport in DRX, comparisons were performed between NMC and Li_1.0_TNO (similar Li content, distinct cation ordering) and between Li_1.2_TNO and Li_1.2_TNMoO (similar Li content, identical structure, different d^0^-TM species). As shown in Fig. 8b, NMC exhibits the highest electronic conductivity due to continuous TM pathways, while all DRX samples exhibit substantially lower conductivity, as insulating d^0^-Ti^4+^ disrupts electron transport. Increasing Li excess further reduces conductivity, whereas Mo substitution moderately enhances electron hopping. Electrochemical measurements reveal distinct kinetic differences between DRX and layered oxides, confirming electronic transport as the rate-determining step for DRX cathodes. As illustrated in Fig. 8c, NMC exhibits high electrochemical activity without carbon coating, whereas DRX (Li_1.0_TNO) becomes electrochemically active only after carbon coating, highlighting its intrinsically poor electronic conductivity. Differential capacity (d*Q*/d*V*) profiles demonstrate that Mo substitution effectively reduces voltage hysteresis relative to Ti-based Li_1.2_TNO. Galvanostatic intermittent titration technique (GITT) results further confirm much larger polarization in DRX than in NMC, verifying that sluggish electron transport dominates the rate-limiting kinetics. Collectively, the intrinsically fragmented electronic network in DRX originates from the random distribution of insulating d^0^-TM units, which induces strong electron localization and ultralow conductivity, ultimately dominating the rate-limiting kinetic behavior of DRX cathodes.

### 3.2 Strategies for enhancing electronic transport

Unlike conventional crystalline materials with well-defined electronic band structures, DRX cathodes lack long-range periodicity, so their electronic structure consists of superimposed local states that induce strong electron localization. This localization arises from two key mechanisms. First, insulating d^0^-TM cations form wide-bandgap (≥2.5 eV) motifs with oxygen, blocking long-range electron migration. Second, even without d^0^ species, redox-active TMs experience strong electron–electron repulsion *via* the Mott–Hubbard effect, further suppressing electron hopping. As a result, electron transport in DRX is dominated by small polaron hopping between isolated TM sites, leading to inherently low conductivity and sluggish kinetics. To address this critical limitation, rational electronic structure engineering has been proposed. Electron doping introduces additional charge carriers to increase carrier density and partially delocalize electronic states.^70^ Among potential dopants, Mo^6+^ is advantageous due to its narrow bandgap, high reducibility, and ability to accommodate extra electrons *via* valence modulation. Highly reductive Mn^2+^ can serve as an effective electron donor. Mechanochemically driven electron transfer from Mn^2+^ to Mo^6+^ enables *in situ* Mo reduction and electron doping within the TM–O framework. This strategy simultaneously enhances intrinsic conductivity and activates reversible Mn^2+^/Mn^4+^ multi-electron redox, improving both charge transport and energy storage. Electron doping thus offers a promising route to mitigate electronic limitations and enable high-rate DRX cathodes.

**Fig. 9:**
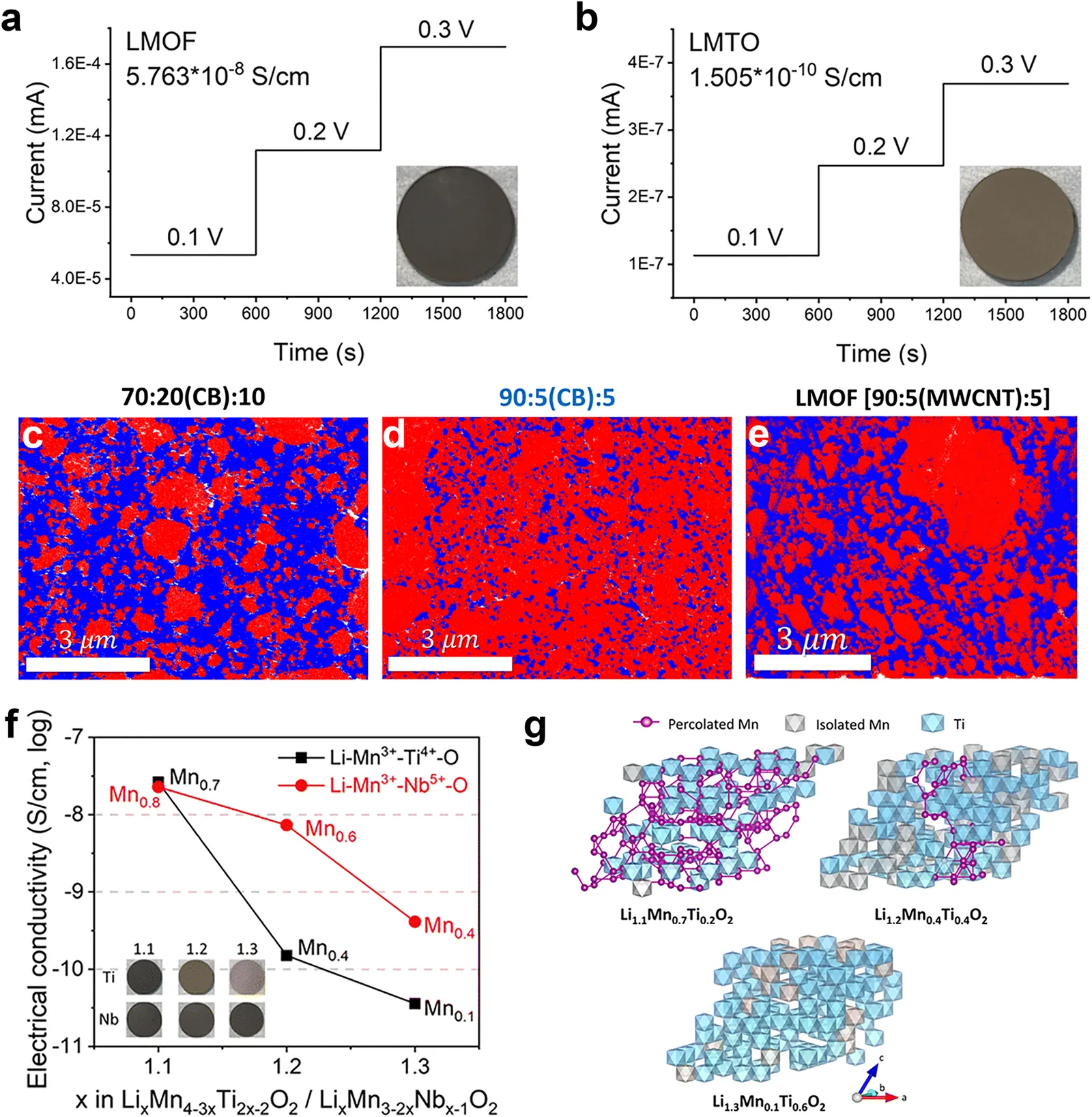
Electrical conductivity and electron transport in Mn-based DRX cathodes. (a and b) DC polarization measurements of the electrical conductivity of LMOF and LMTO pellets. (c and d) SEM-EDS maps of LMOF (red), CB/PVDF (blue), and pore (white) in the 70 : 20 : 10 and 90 : 5 : 5 (LMOF : CB : PVDF) electrodes, respectively. (e) SEM-EDS map of the 90 : 5 : 5 (LMOF : MWCNT : PVDF) electrode, showing LMOF (red), MWCNT/PVDF (blue), and pores (white). (f) Apparent electrical conductivities of Li–Mn–Ti–O and Li–Mn–Nb–O DRX cathodes as a function of Li excess (or Mn content). (g) Representative MC structures for Li_1.1_Mn_0.7_Ti_0.2_O_2_, Li_1.2_Mn_0.4_Ti_0.4_O_2_ and Li_1.3_Mn_0.1_Ti_0.6_O_2_, illustrating the connectivity of the Mn percolation network. Reproduced with permission from ref. 71. Copyright 2024 Royal Society of Chemistry.

Besides intrinsic material modification, electrode engineering has emerged as an effective strategy to enhance the electronic transport of DRX cathodes. Intrinsic bulk conductivity and electrode-level electron transport represent distinct physical phenomena in DRX cathodes that must be clearly differentiated. Intrinsic bulk conductivity arises from small polaron hopping within the disordered cation lattice and is fundamentally governed by the chemical composition and local electronic structure; therefore, its enhancement relies on compositional strategies, such as cation substitution and electronic doping.^18,70^ In contrast, electrode-level electron transport refers to the global electronic conduction through the composite cathode, which is predominantly dictated by carbon coatings, conductive additive networks, electrode formulations, and microstructural contact. Lee *et al.*^71^ demonstrated that the intrinsically low electronic conductivity (10^−10^–10^−8^ S cm^−1^) and severe volume variation of Mn-based DRX materials cause collapse of electrical percolation networks in high-active-material-loading electrodes. As shown in Fig. 9a and b, the electronic conductivities of LMOF and LMTO are only 5.76 × 10^−8^ and 1.51 × 10^−10^ S cm^−1^, respectively, far lower than those of conventional layered oxides and even LiFePO_4_. LMTO conductivity is nearly 380 times lower than that of LMOF. These results indicate that intrinsic conductivity alone is insufficient for high-power operation, necessitating external conductive network design *via* electrode engineering. Replacing conventional carbon black with multi-walled carbon nanotubes (CNTs) offers a viable solution. Owing to their one-dimensional structure and high conductivity, multi-walled CNTs form robust, continuous electron pathways that remain intact under large volume changes, enabling electrodes with ultrahigh active material loading (∼96 wt%). SEM-EDS mapping (Fig. 9c and d) shows that carbon black networks are severely disrupted in high-loading LMOF electrodes, causing increased charge-transfer resistance and rapid degradation. In contrast, multi-walled CNTs construct fully interconnected conductive frameworks even at 90 wt% active material loading (Fig. 9e). These findings confirm that the failure of carbon black-based percolation networks is the main cause of poor high-loading performance for Mn-DRX electrodes, while multi-walled CNTs effectively maintain continuous electron transport.

Further studies reveal that the electronic conductivity of Mn-based DRX strongly correlates with Mn content, with higher Mn fractions favoring improved conductivity. Increasing Li-excess (decreasing Mn content) significantly reduces the conductivity of both Ti- and Nb-based Mn-DRX. At equivalent Li-excess levels, Nb-containing samples exhibit higher conductivity than Ti-based counterparts, due to their higher Mn content (Fig. 9f). Since Ti^4+^ and Nb^5+^ are d^0^ cations and electronically inert, charge transport in Mn-DRX occurs primarily *via* hole hopping within interconnected Mn networks, supported by DFT and Monte Carlo simulations. As illustrated in Fig. 9g, Li_1.1_Mn_0.7_Ti_0.2_O_2_ features a continuous Mn-based conductive network enabling efficient transport. With decreasing Mn content, the network gradually fragments, and Li_1.3_Mn_0.1_Ti_0.6_O_2_ exhibits fully isolated Mn sites, behaving as an insulator. This electrode-level engineering strategy significantly improves electronic transport, rate capability, and cycling stability without altering the intrinsic electronic structure of DRX materials, offering a practical and scalable pathway toward high-energy-density, Ni/Co-free DRX cathodes.

**Fig. 10:**
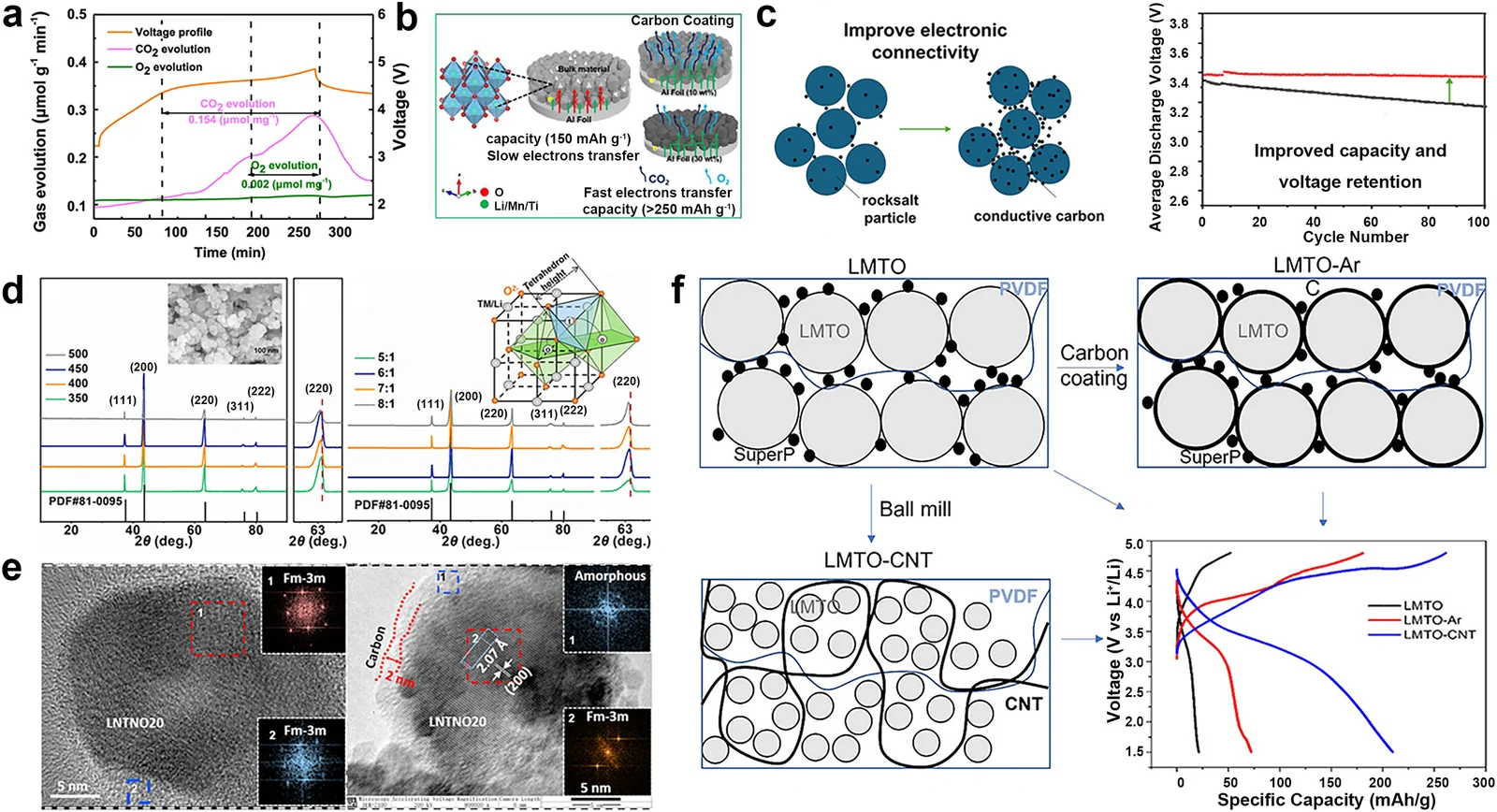
Strategies for enhancing electron transport in DRX cathodes. (a) *Operando* DEMS results for LMTO@C. (b) Schematic diagram of the electron transfer process for the bulk material and the carbon-coating material and gas production process of LMTO@C. Reproduced with permission from ref. 72. Copyright 2019 American Chemical Society. (c) Suppression of capacity and voltage fade in disordered rocksalts by improving electronic contact between particles and reducing oxygen redox. Reproduced with permission from ref. 73. Copyright 2025 Elsevier. (d) XRD results of LNTNO20@C samples using fixed ratio (Li_1.2_Ni_0.3_Ti_0.3_Nb_0.2_O_2_ : sucrose = 6 : 1) calcined at different temperatures and LNTNO20@C samples calcined at 450 °C using different ratios. (e) HRTEM results of bare LNTNO20 and LNTNO20@C with insets of FFT for selected area. Reproduced with permission from ref. 74. Copyright 2021 Elsevier. (f) Enhancing the electrode performance using interfacial carbon deposition and carbon nanotube mediation. Reproduced with permission from ref. 75. Copyright 2023 American Chemical Society.

By constructing a continuous conductive network on the surface of active particles, carbon-based modification strategies can effectively accelerate electron transfer and improve the electrochemical kinetics of DRX cathodes. Such strategies are especially critical for materials with intrinsically low conductivity, such as Mn-based DRX systems and LiFePO_4_. Zhou *et al.*^72^ investigated the degradation of Li_1.2_Mn_0.4_Ti_0.4_O_2_ and revealed that the anionic redox of O^2−^/O_2_^*n*−^ induces irreversible lattice oxygen loss, severe interfacial side reactions, and structural degradation, which increase polarization and cause rapid capacity fading. Carbon coating (10–30 wt%) significantly enhances electrode-level electronic conductivity and redox kinetics, while suppressing O_2_ evolution and CO_2_ generation by 33% and 18%, respectively (Fig. 10a). Coating reduces particle size from ∼10 µm to ∼2 µm and forms a uniform nanoscale carbon layer. As shown in Fig. 10b, this layer establishes continuous electron-transport pathways and mitigates oxygen release and interfacial side reactions, improving cycling stability. Notably, the 30 wt% coated sample delivers nearly twice the capacity retention of the 10 wt% sample after 50 cycles. These results demonstrate that surface carbon coating effectively stabilizes anionic redox and enhances the cycling performance of DRX cathodes.

Similarly, Pi *et al.*^73^ further elucidated the fading mechanisms of DRX cathodes by comparing oxygen-redox-active Li_2_MnO_2_F and oxygen-redox-inactive Li_3_Mn_2_O_3_F_2_. During cycling, an electronically insulating surface layer gradually forms on DRX particles, blocking electron transport between adjacent particles and increasing polarization. Increasing conductive carbon content to 30 wt% constructs a robust conductive network that bypasses the insulating layer, sustains efficient electron transport, and significantly mitigates polarization and voltage decay (Fig. 10c). Mechanistic analysis reveals that oxygen redox dominates the rapid initial capacity and voltage decay, while continuous cathode-electrolyte interphase (CEI) growth and poor interparticle electronic connectivity dominate long-term degradation. These findings confirm that rational electron-transport network engineering is critical for stabilizing DRX cycling performance.

In addition to surface carbon coating, optimizing the conductive carbon architecture in composite electrodes is also critical for improving DRX electrochemistry. Yu *et al.*^74^ synthesized Li_1.2_Ni_0.3_Ti_0.3_Nb_0.2_O_2_@C (LNTNO20@C) using sucrose as the carbon precursor, by optimizing precursor ratio and calcination temperature. Under optimal conditions (LNTNO20 : sucrose = 6 : 1, calcined at 450 °C), the cathode delivers a high initial discharge capacity of 268.2 mAh g^−1^ with an initial coulombic efficiency of 98.3%, and retains 176.4 mAh g^−1^ at 400 mA g^−1^. As shown in Fig. 10d, moderate calcination temperature increases lattice parameters and tetrahedral height, lowering the Li^+^ diffusion barrier and strengthening 0-TM pathways to facilitate ion transport. TEM confirms a uniform amorphous carbon coating on LNTNO20 particles, with the bulk DRX structure well preserved (Fig. 10e). The carbon layer enhances electrode-level electronic conductivity, suppresses polarization, mitigates interfacial side reactions, and improves structural stability.

To further address the low conductivity and poor electrode-level capacity of Li_1.2_Mn_0.4_Ti_0.4_O_2_ (LMTO), vapor-phase amorphous carbon deposition and CNT-assisted ball milling have been developed.^75^ Conventional Super P carbon black is discretely distributed among LMTO grains, failing to form continuous electron pathways (Fig. 10f), which leads to severe polarization and a discharge capacity of only ∼50 mAh g^−1^. Depositing amorphous carbon *via* C_2_H_2_ precursor increases conductivity by approximately 5 orders of magnitude, but large irreversible capacity loss limits the first discharge to 70 mAh g^−1^ despite a high first charge capacity of 180 mAh g^−1^. In contrast, ball milling with multi-walled CNTs builds a robust three-dimensional conductive framework, enabling a high active-material loading of 78.7 wt%. The LMTO-CNT electrode delivers a first-cycle discharge capacity of 165 mAh g^−1^, exceeding the 155 mAh g^−1^ of the 20 wt% SuperP-based electrode. These results indicate that while carbon coating improves particle conductivity, poor interparticle contact and surface-related side reactions still cause irreversible loss. By comparison, interconnected CNT networks simultaneously enable high loading, efficient electron transport, and improved structural stability, effectively activating the intrinsic capacity and reducing polarization.^76,77^

Because DRX cathodes require a high conductive carbon content (up to 30 wt%) for efficient active-material utilization, the resulting reduction in cell-level energy density remains a major challenge. To address this issue, Patil *et al.*^78^ investigated the effects of carbon microstructure and loading (10–20 wt%) on the electrochemical behavior of DRX cathodes. Unlike conventional amorphous carbon additives (Super C65 and Ketjenblack), which cause rapid capacity fading *via* unstable cathode-electrolyte interphases, a DRX cathodes with only 10 wt% graphite exhibits significantly improved cycling stability and rate capability. This superior performance arises from the uniform graphitic coating on DRX particles, which suppresses high-voltage parasitic reactions, and the homogeneous graphite dispersion, which maintains a robust, stable conductive network during cycling. Collectively, these results demonstrate that carbon-based conductive engineering provides an effective and scalable strategy for simultaneously improving electrode-level electron transport, interfacial stability, and electrochemical performance in DRX cathodes.

## 4 Design principles for high-performance DRX

Despite the remaining challenges associated with practical implementation, DRX cathodes offer a rare combination of high capacity, broad compositional flexibility, low cost, and elemental abundance. In principle, they can approach the energy density of state-of-the-art layered oxides while offering sustainability and affordability advantages associated with Co/Ni-lean or Co/Ni-free chemistries. The design principles provide a framework for developing high-performance DRX cathodes by jointly optimizing ionic transport, electronic transport, local structure, and interfacial stability.

### 4.1 Balancing Li^+^ percolation and electronic conductivity

Early DRX design was guided primarily by the 0-TM percolation theory, in which increased Li-excess (*x* > 1.1 in Li_*x*_TM_2−*x*_O_2_) generated more 0-TM channels and accelerated Li^+^ diffusion. Recent studies, however, indicate that high-performance DRX cathodes require simultaneous optimization of Li^+^ percolation and electronic conductivity. Li-excess should therefore be maintained within an appropriate range. Low Li-excess (approximately 1.0–1.05) preserves more continuous redox-active TM networks and favors electronic conductivity, but limits 0-TM connectivity and Li^+^ diffusion. In contrast, high Li-excess (≥1.3) improves Li^+^ transport but dilutes the TM network and weakens electronic conduction. This trade-off is particularly pronounced in Mn-based DRX cathodes, where conductivity depends strongly on Mn content. In addition to electronic conductivity penalties, excessive Li-excess also brings practical drawbacks for electrode fabrication. High Li-excess not only compromises electronic conductivity through reduced Mn content, but also exacerbates voltage hysteresis *via* enhanced oxygen redox activity.^9,73^ Together, these effects increase the demand for conductive carbon loading, which ultimately reduces the cell-level practical energy density. Notably, in Mn-rich DRX systems, the formation of the δ phase simultaneously balances Li^+^ percolation and electronic conductivity, since the δ phase facilitates Li^+^ transport while the high Mn content ensures adequate electronic conduction. Therefore, rather than maximizing Li content, the compositional design of DRX cathodes should seek a balanced Li-excess level that reconciles ionic and electronic transport while preserving practical electrode-level performance.

Table 1 summarizes representative electrochemical performance, Li^+^ diffusivity, and electronic conductivity data for DRX cathodes reported in literature. It should be noted that these transport metrics were measured *via* diverse characterization techniques, such as GITT, PITT, and EIS for Li-ion diffusion, while DC polarization and four-probe measurements for electronic conductivity. Because of these methodological variations, direct quantitative comparisons across different materials should be approached with particular caution. Nevertheless, the overarching performance trends in Table 1 indicate that maintaining a balanced Li excess, rather than maximizing Li excess, is vital for concurrently optimizing capacity, rate capability, and practical electrode level energy density.

**Table 1:** Electrochemical performance, ionic diffusivity and electronic conductivity of DRX cathodes

Materials	Li^+^ diffusivity (cm^2^ s^−1^)	Electronic conductivity (S cm^−1^)	Measurement method	Active material : carbon : binder	Initial capacity (mAh g^−1^)	Rate (mAh g^−1^)	Ref.
Li_1.2_Mn_0.4_Ti_0.4_O_2_ (LMTO)	10^−15^–10^−14^	1.51 × 10^−10^	PITT; DC polarization	7 : 2 : 1	260 at 10 mA g^−1^ (1.5–4.7 V)	∼191 at 260 mA g^−1^	28 and 70
Li_1.2_Mn_0.4_Zr_0.4_O_2_ (LMZO)	10^−16^–10^−15^	—	PITT		∼171 at 10 mA g^−1^ (1.5–4.7 V)	∼82 at 260 mA g^−1^	
Li_1.3_Nb_0.3_Mn_0.4_O_2_ (LNMO)	∼3.2 × 10^−12^	2.27 × 10^−6^	GITT; DC polarization	6 : 3 : 1	∼256 at 10 mA g^−1^ (1.8–4.6 V)	81.3 at 500 mA g^−1^	60
Li_1.3032_Mn_0.398_Nb_0.3018_O_1.9516_S_0.047_ (LNMOS-32)	∼7.6 × 10^−12^	3.82 × 10^−6^			306 at 10 mA g^−1^ (1.8–4.6 V)	123.8 at 500 mA g^−1^	
Li_1.05_Mn_0.90_Nb_0.05_O_2_ (M90)	10^−16^–10^−15^	1.8 × 10^−4^	GITT; four point probe	7 : 2 : 1	261 at 10 mA g^−1^ (1.5–4.8 V)	185 at 400 mA g^−1^	25
Li_1.20_Mn_0.60_Nb_0.20_O_2_ (M60)	10^−15^–10^−14^	8.6 × 10^−5^			287 at 10 mA g^−1^ (1.5–4.8 V)	189 at 400 mA g^−1^	
Li_1.2_Mn_0.2_Ti_0.4_Cr_0.2_O_2_ (LMTC02O)	4 × 10^−16^	—	GITT	7 : 2 : 1	257 at 20 mA g^−1^ (1.5–4.8 V)	155 at 1000 mA g^−1^	64
Li_1.2_Ni_0.2_Ti_0.6_O_2_ (LNTO)	∼1 × 10^−17^				216 at 20 mA g^−1^ (1.5–4.6 V)	64 at 1000 mA g^−1^	
Li_1.2_Ni_0.1_Ti_0.5_Cr_0.2_O_2_ (LNTC02O)	∼1 × 10^−16^				271 at 20 mA g^−1^ (1.5–4.6 V)	160 at 1000 mA g^−1^	
LiNi_0.5_Ti_0.5_O_2_ (LNTO)	8.51 × 10^−15^	—	EIS	8 : 1 : 1	120 at 20 mA g^−1^ (1.5–4.5 V)	∼80 at 400 mA g^−1^	79
Li_1.1_Ni_0.4_Ti_0.4_Nb_0.1_O_2_ (LNTO10)	4.69 × 10^−14^				181.7 at 20 mA g^−1^ (1.5–4.5 V)	∼100 at 400 mA g^−1^	
Li_1.2_Ni_0.3_Ti_0.3_Nb_0.2_O_2_ (LNTO20)	9.35 × 10^−14^				221.5 at 20 mA g^−1^ (1.5–4.5 V)	∼125 at 400 mA g^−1^	
Li_1.2_Ni_0.3_Ti_0.3_Nb_0.2_O_2_ (LNTNO20)@C	5.568 × 10^−13^	—	EIS	8 : 1 : 1	268.2 at 20 mA g^−1^ (1.5–4.5 V)	176.4 at 400 mA g^−1^	74
Li_1.47_Mn_1.6_O_3.7_F_0.3_	10^−14^–10^−12^	—	GITT	7 : 2 : 1	350 at 50 mA g^−1^ (1.5–4.8 V)	158 at 7500 mA g^−1^	55
Li_1.05_Mn_0.85_Ti_0.1_O_2_ (ADRX-LTMO)	10^−14^–10^−12^	—	GITT	7 : 2 : 1	281 at 50 mA g^−1^ (1.5–4.8 V)	153 at 2000 mA g^−1^	49
Li_1.2_Mn_0.55_Ti_0.25_O_1.85_F_0.15_ (LMTF-4 h)	10^−18^–10^−16^	—	GITT	6 : 3 : 1	273 at 20 mA g^−1^ (1.5–4.8 V)	101 at 2000 mA g^−1^	80
Li_1.2_Mn_0.55_Ti_0.25_O_1.85_F_0.15_ (LMTF-35 min)	10^−17^–10^−15^				313 at 20 mA g^−1^ (1.5–4.8 V)	143 at 2000 mA g^−1^	
Li_2_MnO_2_F	10^−12^–10^−11^	—	GITT	8 : 1 : 1/6 : 3 : 1	254 at 20 mA g^−1^ (2–4.8 V)	—	73
Li_3_Mn_2_O_3_F_2_					221 at 20 mA g^−1^ (2–4.8 V)		
Li_1.2_Mn_0.4_Ti_0.4_O_2_ (LMTO, Joule-heating-10 min)	∼8.1 × 10^−15^	—	GITT	7 : 2 : 1	230.6 at 20 mA g^−1^ (1.5–4.8 V)	161 at 1000 mA g^−1^	34
Li_1.68_Mn_1.6_O_3.7_F_0.3_ (LMOF)	—	5.76 × 10^−8^	DC polarization	7 : 2 : 1	∼370 at 25 mA g^−1^ (1.5–4.8 V)	∼325 at 250 mA g^−1^	70
Li_1.0_Ti_0.5_Ni_0.5_O_2_ (Li_1.0_TNO)	—	3.54 × 10^−7^	DC polarization	6 : 3 : 1	150 at 10 mA g^−1^ (1.5–4.8 V)	—	18 and 79
Li_1.1_Ti_0.55_Ni_0.35_O_2_ (Li_1.1_TNO)		1.02 × 10^−7^			∼175 at 10 mA g^−1^ (1.5–4.8 V)		
Li_1.2_Ti_0.6_Ni_0.2_O_2_ (Li_1.2_TNO)		1.14 × 10^−9^			∼175 at 10 mA g^−1^ (1.5–4.8 V)		
Li_1.2_Ti_0.333_Ni_0.33_Mo_0.13_O_2_ (Li_1.2_TNMoO)		1.13 × 10^−7^			∼260 at 10 mA g^−1^ (1.5–4.8 V)		

### 4.2 Rational selection of d^0^ TM

The proper selection of d^0^ TMs constitutes another key design strategy for high-performance DRX cathodes. Redox-inactive species such as Ti^4+^, Nb^5+^, Mo^6+^, Zr^4+^, and Ta^5+^ help stabilize long-range cation disorder. Their ionic radii and electrostatic interactions with Li^+^ determine local SRO and therefore affect the number and connectivity of 0-TM channels. Smaller d^0^ cations such as Ti^4+^ (0.605 Å) show a considerable ionic-radius mismatch with Li^+^ (0.76 Å), which can promote Li-rich local environments and facilitate 0-TM channel formation. In contrast, larger d^0^ species such as Zr^4+^ (0.72 Å) have radii closer to that of Li^+^, favoring more homogeneous cation distributions and Li_3_M-type local configurations associated with 1-TM channels and higher Li^+^ migration barriers. Accordingly, Li^+^ transport in DRX cathodes is governed not only by the long-range average structure but also by local SRO and cation chemistry.

The choice of d^0^ TM also strongly influences electronic conduction. Insulating d^0^-octahedral frameworks interrupt electron-hopping pathways between redox-active TM sites. Ti^4+^ is widely used because of its low cost, natural abundance, and ability to stabilize DRX lattices while promoting Li^+^ percolation. Mo^6+^, in contrast, offers electronic advantages because the relatively narrow Mo 4d–O 2p energy separation can enhance orbital hybridization and partially alleviate electron localization. Nevertheless, excessive incorporation of any d^0^ species dilutes the concentration of redox-active TMs, disrupts TM–TM connectivity, and degrades electronic conductivity. Therefore, both the type and concentration of d^0^ TMs must be optimized to maintain structural stability, enhance Li^+^ percolation, preserve electronic pathways, and mitigate oxygen-redox and interfacial instabilities.

### 4.3 Balancing cation disorder and local order

Although long-range cation disorder is needed to form continuous 0-TM Li^+^ diffusion pathways, fully disordered DRX materials often suffer from sluggish electrochemical kinetics, severe electron localization, and low electronic conductivity. In contrast, spinel oxides exhibit fast three-dimensional Li^+^ diffusion and superior kinetic properties. The formation of spinel-like δ phases in Mn-rich DRX cathodes (δ-DRX) can synergize the advantages of ordered and disordered structures. Specifically, δ-DRX retains sufficient disorder to preserve Li-rich diffusion networks while introducing local spinel-like ordering that improves transport connectivity and suppresses deleterious two-phase reactions. As a result, partially ordered DRX materials generally exhibit better kinetic performance than fully disordered DRX. Developing scalable, low-cost, and controllable synthetic or electrochemical activation methods to stabilize δ-like local ordering remains an important future challenge. More broadly, order–disorder modulation provides a useful strategy for designing high-performance Mn-based cathodes that combine fast transport, structural stability, and low cost.

## 5 Perspectives

**Fig. 11:**
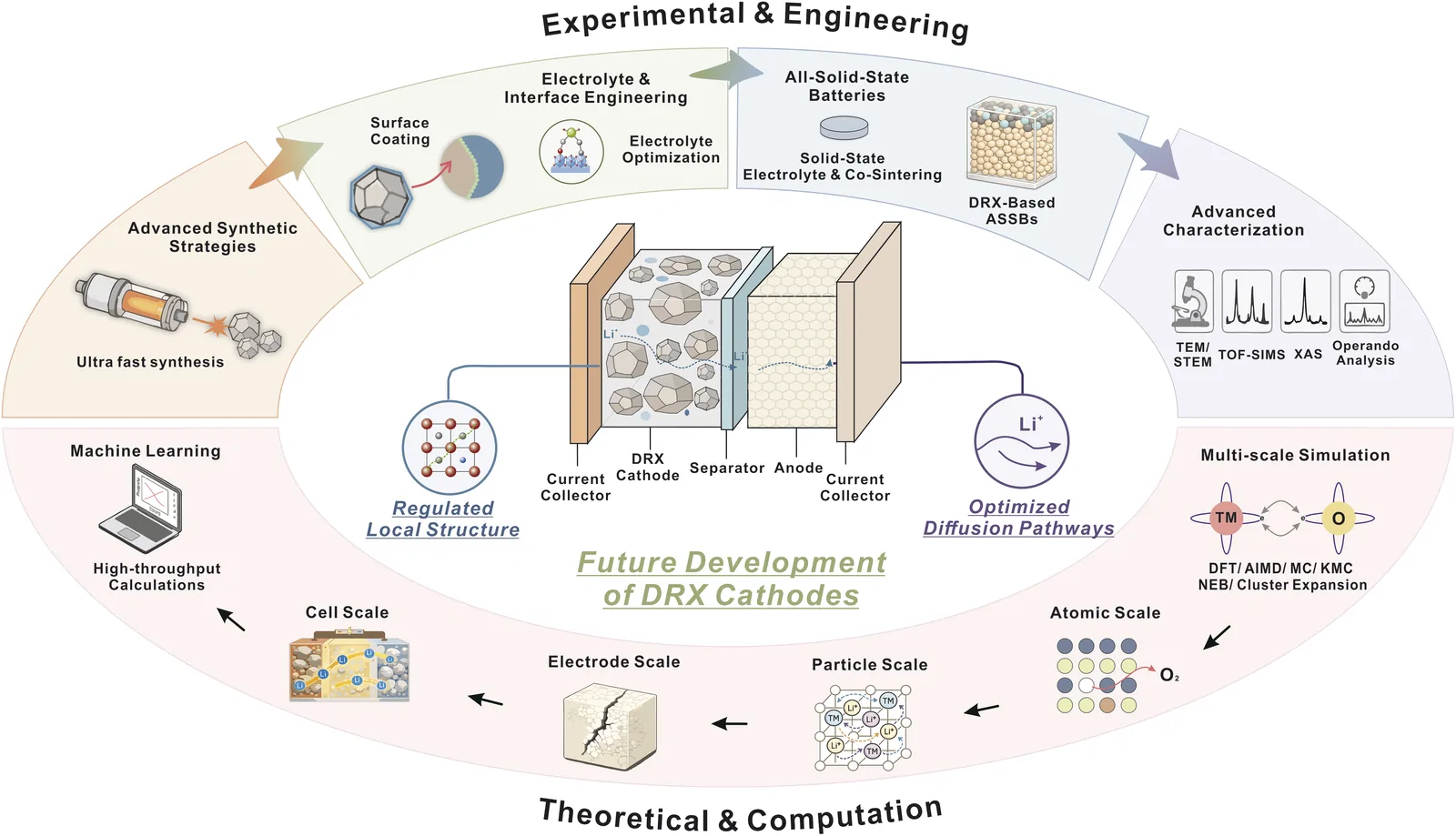
Expected future directions for the development of DRX cathodes.

As summarized in Fig. 11, future research on DRX cathodes needs to focus on advanced non-equilibrium synthesis, electrolyte and interface engineering, DRX-based all-solid-state batteries, advanced characterization, and machine-learning-assisted materials design. These directions provide opportunities to precisely regulate local structure, interfacial chemistry, and electrochemical reaction pathways, thereby accelerating the development of high-energy-density and long-cycle-life DRX cathodes.

### 5.1 Advanced synthetic strategies for non-equilibrium and kinetic control

Currently, most DRX cathodes are synthesized *via* conventional high-temperature solid-state reactions, which require prolonged annealing at ∼1000 °C for over 12 h to obtain phase-pure cation-disordered structures. However, these processes are both energy- and time-intensive and provide limited control over particle size, morphology, compositional homogeneity, and local structural ordering, often resulting in large particles (10–40 µm). Extended high-temperature treatment can further induce unfavorable SRO, grain coarsening, and elemental segregation, all of which are detrimental to Li^+^ transport kinetics and electrochemical performance. Therefore, the future development of high-performance DRX cathodes will increasingly rely on advanced non-equilibrium synthetic strategies.^81^ To date, various non-equilibrium synthetic approaches, including mechanochemical synthesis, rapid quenching, and ultrafast thermal treatments (*e.g.*, microwave-assisted and Joule-heating synthesis), have been explored for DRX and high-entropy cathodes. Among them, mechanochemical synthesis promotes atomic-scale elemental mixing and room-temperature disordering, facilitating the formation of highly cation-disordered structures and enabling fluorine incorporation beyond the thermodynamic solubility limit. Rapid thermal processing and quenching can effectively freeze high-temperature metastable states and suppress Li/TM reordering during cooling. Meanwhile, ultrafast heating enables rapid phase formation and controllable defect engineering within extremely short processing times while minimizing grain coarsening and elemental segregation. Collectively, non-equilibrium kinetic processes play a critical role in regulating the local structure and electrochemical behavior of DRX materials.

Nevertheless, the non-equilibrium synthesis of DRX materials still faces several critical challenges. First, the formation mechanisms of cation disorder, SRO, and defect configurations under different non-equilibrium conditions remain poorly understood, and a systematic framework correlating synthetic parameters, local structure, and electrochemical performance is still lacking. Second, many kinetically trapped metastable structures may undergo gradual ordering or phase separation during electrochemical cycling, and their thermodynamic stability and structural evolution pathways require further clarification. Furthermore, most current non-equilibrium strategies still rely heavily on empirical optimization, and a unified theoretical model describing the roles of heating rate, cooling rate, diffusion kinetics, and structural freezing has not yet been established. Future research needs to integrate advanced *in situ* characterization, machine learning, and thermodynamic/kinetic simulations to develop multiscale structure-evolution models, thereby enabling precise and predictable control over non-equilibrium synthesis. In parallel, the development of low-cost, scalable, and industrially compatible synthetic routes will be essential for translating high-performance DRX and high-entropy cathodes from laboratory research to practical battery applications.

### 5.2 Electrolyte and interface engineering

Electrolytes play a crucial role in regulating the interfacial chemistry and electrochemical performance of DRX cathodes. Although some lithium salts, solvents, and additives have proven effective for Li-rich layered oxides, their applicability to DRX systems remains insufficiently explored. Conventional low-concentration LiPF_6_-carbonate electrolytes exhibit limited oxidation stability (<4.3 V), which cannot satisfy the high operating voltage (4.8 V) required to fully activate DRX cathodes.^82,83^ Consequently, severe electrolyte decomposition, gas evolution, unstable CEI formation, and parasitic interfacial reactions occur during cycling. These issues are further aggravated by the irreversible oxygen release associated with anionic redox in DRX materials, ultimately accelerating interfacial degradation and capacity fading. Therefore, the development of advanced electrolytes with tailored solvation structures is essential for suppressing parasitic reactions and stabilizing electrode–electrolyte interfaces.

High-concentration electrolytes (HCEs) have shown considerable potential for improving the reversibility of DRX cathodes. By significantly reducing free solvent molecules, HCEs suppress transition-metal dissolution and interfacial side reactions. For example, concentrated LiFSA:DMC and LiFSI-based electrolytes stabilize the CEI and significantly improve cycling stability.^84–87^ In addition, combining HCEs with aramid-coated separators further stabilizes anionic redox and mitigates oxygen loss in Mn-based DRX cathodes. Fluorinated oxyfluorides such as Li_2_MnO_1.5_F_1.5_, when paired with HCEs, also exhibit stable cycling without obvious voltage decay.^88,89^ Nevertheless, the high viscosity, poor wettability, and elevated cost of HCEs continue to hinder their practical application. Localized high-concentration electrolytes, formed by introducing non-coordinating diluents into HCEs, have emerged as promising alternatives because they combine high oxidative stability with lower viscosity and reduced cost. Their optimized solvation structures promote the formation of uniform and robust interphases, suppress electrolyte decomposition, and facilitate efficient Li^+^ transport.^90^ Further optimization of solvent compositions enables controlled solvation structures with limited free solvent molecules and abundant ion aggregates, resulting in high oxidative stability (∼5.1 V) and robust interphases that suppress particle cracking, Mn dissolution, and gas evolution, thereby markedly extending cycle life.^91^ Electrolyte design has also been extended to fast-charging applications, where the DMC content plays a decisive role in balancing rate capability and cycling stability. High DMC concentrations favor rapid Li^+^ transport and superior rate performance, whereas lower DMC contents promote more stable interfacial chemistry and longer cycling life.^92^ These findings highlight the importance of simultaneously balancing ionic transport, interfacial stability, and structural robustness in DRX cathodes systems.

Ionic liquids have also emerged as promising electrolytes for DRX cathodes owing to their low chemical reactivity and excellent oxidative stability. Compared with conventional carbonate electrolytes, ionic liquids-based electrolytes can effectively suppress parasitic reactions induced by reactive oxygen species released from DRX cathodes, thereby mitigating electrolyte decomposition and stabilizing the CEI. Conventional carbonate electrolytes typically form thick and unstable LiF-/Li_2_CO_3_-rich CEI layers, whereas ionic liquids electrolytes favor the formation of thin and robust interphases that preserve anionic redox activity, suppress gas evolution, improve coulombic efficiency, and alleviate long-term capacity fading.^93^ Solvate ionic liquids, such as [Li(G3)] [TFSI] and diluted solvate ionic liquids systems, exhibit superior chemical inertness toward superoxide-like reactive species, enabling more stable electrode–electrolyte interfaces, reduced polarization, and improved reversibility of anionic redox reactions. Recent studies have further identified cation densification and cathode overcharge as key degradation pathways in DRX cathodes, highlighting the critical role of electrolyte engineering in stabilizing interfacial chemistry and sustaining long-term electrochemical performance.^94^ Future efforts should focus on developing low-viscosity ionic liquids/solvate ionic liquids systems with enhanced ionic conductivity, wider operating-temperature windows, and improved compatibility with high-capacity DRX cathodes.

Electrolyte additives provide an efficient and cost-effective strategy for mitigating high-voltage interfacial instability and capacity fading in Li-rich DRX cathodes. Conventional additives, such as vinylene carbonate and fluoroethylene carbonate are widely used commercial SEI/CEI-forming additives; however, their effectiveness in DRX systems is limited. Vinylene carbonate tends to undergo premature oxidation at ∼4.6 V, triggering additional side reactions and accelerating capacity degradation. Although fluoroethylene carbonate can improve coulombic efficiency, its contribution to long-term capacity retention is limited, restricting its applicability to relatively low-voltage DRX systems.^95^ Currently, silicon-based tris(trimethylsilyl) phosphite (TMSPI), borate-based lithium difluoro(oxalato)borate (LiODFB), and phosphate-based additives have demonstrated considerable potential for improving cycling stability and rate capability by scavenging acidic impurities, suppressing transition-metal dissolution, and facilitating the formation of stable CEI layers.^96,97^ Nevertheless, several critical challenges remain unresolved, including unclear interfacial reaction mechanisms, limited compatibility with different DRX chemistries, and insufficient long-term stability under high-voltage and elevated-temperature conditions. Future research needs to therefore focus on the rational design of multifunctional composite additives with improved interfacial adaptability and synergistic effects. Moreover, combining advanced *in situ*/*operando* characterization techniques with theoretical simulations will be essential for elucidating interfacial evolution mechanisms and establishing structure–function relationships to guide additive design for diverse DRX systems.

Surface coating is another effective strategy for improving the interfacial stability of DRX cathodes. Ultrathin inorganic coatings, such as Al_2_O_3_ and B_2_O_3_ deposited *via* atomic layer deposition, can uniformly cover DRX particle surfaces, thereby suppressing oxygen release and parasitic electrolyte reactions, reducing interfacial impedance, and enhancing rate capability.^98,99^ However, inorganic coatings often exhibit strong thickness dependence and are susceptible to mechanical degradation during prolonged cycling, limiting their ability to mitigate bulk structural deterioration. Future research needs to target multifunctional composite coatings that integrate conductive polymers with inorganic materials to simultaneously achieve high ionic/electronic conductivity and robust structural stability. In addition, emerging strategies such as doped coatings, gradient coatings, and self-adaptive interfacial layers warrant deeper exploration to optimize interfacial ion/electron transport kinetics. Ultimately, rational electrolyte design, interfacial engineering, and surface modification will be indispensable for overcoming the intrinsic instability of DRX cathodes and enabling their practical application in next-generation high-energy lithium-ion batteries.

### 5.3 DRX-based all-solid-state batteries

While Mn-based DRX cathodes (including HE-DRX) demonstrate high capacity in liquid electrolyte systems, their long-term cycling stability is often limited by electrolyte-induced interfacial reactions and CEI instability. Transitioning to all-solid-state batteries (ASSBs) provides an opportunity to alleviate liquid-electrolyte-related degradation;^100–102^ however, the electrochemical performance of ASSBs is strongly governed by the chemical compatibility, mechanical stability, and interfacial contact between cathode materials and solid electrolytes. Unlike liquid electrolytes that can effectively infiltrate porous cathode structures and provide continuous ionic pathways, solid electrolytes rely on solid–solid contact, where insufficient interfacial contact, poor wettability, and mechanical degradation during cycling can result in sluggish Li^+^ transport and increased interfacial resistance. Therefore, constructing stable cathode/solid electrolyte interfaces and integrated composite cathode architectures by combining active materials, solid electrolytes, conductive additives, and interfacial modifiers has emerged as an effective strategy to establish continuous ionic/electronic transport networks and enhance the electrochemical performance of DRX cathodes in ASSBs.

Compared with conventional layered oxide cathodes (*e.g.*, NCM, NCA, and LCO), DRX materials possess several structural advantages for integrated solid-state electrode design. Layered cathodes generally experience significant lattice variation (∼10%) during repeated lithiation/delithiation, leading to mechanical stress accumulation, interfacial void formation, and rapid capacity degradation.^103,104^ In contrast, several DRX compositions exhibit ultralow volume variation (0.02–1.44%), effectively mitigating mechanical degradation at solid–solid interfaces.^5,105^ For example, Li_1.211_Mo_0.467_Cr_0.3_O_2_ (LMCO) exhibits only a negligible volume change of 0.12% and maintains excellent interfacial mechanical stability when coupled with amorphous Li_2_S–P_2_S_5_ (LPS) sulfide electrolyte, demonstrating the potential of zero-strain DRX cathodes for suppressing mechanical failure in ASSBs.^106^ Beyond mechanical stability, the compositional flexibility and disordered framework of DRX materials provide additional opportunities for tailoring cathode/electrolyte compatibility and constructing integrated electrode architectures.

Garnet-type oxide electrolytes, particularly Li_6.4_La_3_Zr_1.4_Ta_0.6_O_12_ (LLZTO), have attracted considerable attention for ASSBs owing to their high ionic conductivity, excellent chemical stability, and mechanical robustness. However, achieving intimate cathode/LLZTO interfaces remains challenging due to poor solid–solid wettability and high interfacial resistance.^107,108^ Conventional layered oxide cathodes, such as LCO, often suffer from severe elemental interdiffusion and secondary phase formation with LLZTO during high-temperature processing, whereas low-temperature fabrication generally results in insufficient interfacial contact. In this regard, DRX-based composite cathodes combined with co-sintering strategies provide a promising pathway for constructing robust cathode/garnet interfaces. In particular, HE-DRX cathodes coupled with ultrafast high-temperature sintering utilize entropy stabilization to suppress interfacial reactions, while rapid densification minimizes elemental diffusion and enables the formation of dense and conformal cathode/electrolyte interfaces. Such integrated architectures significantly reduce interfacial impedance and highlight the potential of DRX cathodes for garnet-based ASSBs.^109^

Beyond oxide electrolytes, sulfide electrolytes have also been widely investigated in DRX-based ASSBs due to their high room-temperature ionic conductivity (∼10^−2^ S cm^−1^). Nevertheless, their application is hindered by interfacial instability and parasitic reactions with cathode components. Although conductive carbon additives are essential for improving the electronic conductivity of DRX electrodes, excessive carbon loading may promote parasitic reactions with sulfide electrolytes and accelerate interfacial degradation. To address these issues, surface modification strategies have been explored. For example, Li_3_BO_3_ (LBO) nanocoating can suppress Li_6_PS_5_Cl (LPSCl) decomposition and reduce interfacial resistance. Compared with LiNbO_3_ (LNO) and Li_2_ZrO_3_ (LZO) coatings, LBO-modified DRX cathodes exhibit improved capacity and cycling stability, demonstrating the importance of interfacial engineering.^110^

Halide electrolytes, such as Li_3_InCl_6_ (LIC) have attracted increasing attention because of their high oxidative stability and wide electrochemical windows.^111,112^ Their favorable chemical compatibility with high-voltage cathodes enables reduced interfacial degradation and improved cycling performance. However, further optimization of electrolyte composition, cathode/electrolyte compatibility, and interfacial regulation strategies remains necessary to fully exploit their potential in DRX-based ASSBs. Future advances in all-solid-state batteries will focus on the synergistic integration of zero-strain/HE-DRX cathodes, optimized sulfide/halide electrolytes, nanocoating strategies, and oxide electrolyte–cathode co-sintering technologies. Resolving both chemical and mechanical interfacial instability will be critical to advancing DRX-based all-solid-state batteries toward high energy density, long cycle life, and practical deployment.

### 5.4 Advanced characterization

Advanced characterization techniques will play an increasingly important role in unraveling the complex electrochemical behavior and degradation mechanisms of DRX cathodes. Owing to intrinsic cation disorder, local structural heterogeneity, and coupled cationic/anionic redox processes, conventional characterization methods are often insufficient to capture the dynamic structural and chemical evolution during cycling. Recently, advanced characterization techniques, including synchrotron X-ray diffraction (XRD), X-ray absorption spectroscopy (XAS), neutron pair distribution function (nPDF), solid-state nuclear magnetic resonance (ssNMR), (scanning) transmission electron microscopy (TEM/STEM), atom probe tomography (APT), and differential electrochemical mass spectrometry (DEMS), have provided critical insights into Li-ion transport, local structural evolution, redox chemistry, interfacial degradation, and gas-release mechanisms.^113–115^Table 2 summarizes the major characterization techniques applied to DRX cathodes and their corresponding research applications. Among these techniques, STEM-based methods, nPDF, and ssNMR have enabled direct visualization and quantitative characterization of cationic SRO, providing important insights into Li distribution, percolation networks, and the influence of cation chemistry on local structural environments.^13,30,116^ In contrast, studies on anionic SRO remain relatively limited. Advanced techniques with high sensitivity toward light-element, such as ptychography-STEM and nPDF, are expected to further clarify the effects of anion ordering on Li diffusion pathways and percolation networks. Meanwhile, ^7^Li ssNMR, ToF-SIMS, XCT imaging, and especially APT have demonstrated strong potential for probing local Li environments and three-dimensional elemental distributions.^117–119^ Notably, APT enables 3D atomic-scale reconstruction of Li and transition-metal distributions, offering unique insights into local compositional heterogeneity, Li percolation networks, and nanoscale structural evolution.^120–122^ Advanced characterization methods have also significantly improved the understanding of oxygen redox chemistry and degradation mechanisms in DRX cathodes. Techniques such as ptychography-STEM, resonant inelastic X-ray scattering (RIXS), ssNMR, and nPDF can reveal oxygen evolution behavior and the associated structural degradation at both bulk and surface levels.^123,124^ Moreover, spatially resolved techniques such as TXM-XAS and STXM-XAS enable visualization of redox heterogeneity and charge distribution within individual DRX particles, revealing particle size-dependent reaction kinetics and heterogeneous redox behavior between particle surfaces and interiors. Complementary techniques, including XAS, ToF-SIMS, and APT, provide valuable insights into transition-metal evolution, CEI composition, and interfacial chemical heterogeneity.

**Table 2:** Advanced characterization techniques and key scientific issues in DRX cathodes

Characterization techniques	Main information obtained	Applications in DRX cathode	Ref.
STEM	Atomic-scale structural information, elemental distribution, and local atomic arrangements	Reveals SRO and local structural evolution in DRX materials	125 and 126
nPDF	Local atomic coordination, interatomic distances, and short-range structural information	Provides insights into Li/TM/O local environments	28
ssNMR	Local chemical environments of light elements (*e.g.*, Li, O, F)	Probes Li distribution and local structural evolution	127
RIXS	Electronic structure and oxygen-related states	Investigates oxygen redox activation, oxygen hole formation, and the reversibility of anionic redox reactions	36
XAS	TMs oxidation states and local coordination environments	Tracks TMs redox, electronic structure evolution, and local chemical changes during cycling	128
STXM-XAS/TXM-XAS	Spatially resolved chemical states, oxidation-state distributions, and redox heterogeneity	Maps TMs valence distribution across nanoscale to particle scale; unravels particle-size-dependent redox kinetics, charge heterogeneity and uneven electrochemical reactions	48 and 129
APT	33D atomic-scale elemental distribution	Provides direct visualization of Li/TM spatial distribution, elemental heterogeneity, Li percolation networks, and nanoscale structural evolution	130
ToF-SIMS	Depth-resolved chemical composition and spatial distribution of ionic species	Analyzes CEI composition, interfacial reaction products, and Li distribution across interfaces	131
XCT	3D morphology and structural information	Monitors particle cracking, pore formation, and structural degradation during cycling	132
DEMS	Gas evolution behavior during electrochemical cycling	Quantifies oxygen release, electrolyte decomposition, and parasitic reactions	133
Synchrotron XRD	Long-range crystal structure, lattice parameters, and phase evolution	Monitors phase transformation, lattice variation, and structural stability during charge/discharge	46

In parallel with experimental characterization, multiscale computational modeling and machine learning-assisted high-throughput calculations are emerging as powerful tools for accelerating the rational design of DRX cathodes. Due to the vast compositional space and complex local disorder in DRX systems, conventional trial-and-error experiments and isolated first-principles calculations are insufficient to establish comprehensive structure–property relationships. Multiscale modeling frameworks integrating atomic-scale simulations, particle-level models, electrode-scale electrochemical models, and cell-level simulations provide an effective approach for bridging local atomic structures with macroscopic battery performance.^134,135^ At the atomic level, density functional theory (DFT) and Monte Carlo simulations can elucidate Li-ion migration mechanisms, SRO evolution, phase stability, and redox behavior, while mesoscale and continuum models can predict particle-level reaction heterogeneity, mechanical degradation, and transport limitations.

Furthermore, integrating high-throughput calculations, machine learning algorithms, and advanced characterization datasets offers new opportunities for accelerating material discovery and optimization. These data-driven strategies can rapidly predict compositional stability, Li diffusion pathways, SRO tendencies, and electrochemical properties across broad chemical spaces, enabling the establishment of quantitative processing–structure–property relationships. Despite significant progress, several fundamental challenges remain unresolved, including the dynamic evolution of SRO, the reversibility of oxygen redox reactions, and the coupling between bulk structural degradation and interfacial instability under realistic operating conditions. Future studies should focus on integrating multimodal *in situ*/*operando* characterization with multiscale simulations and machine learning to achieve predictive understanding of structural evolution and electrochemical behavior in DRX cathodes. Moreover, characterization and modeling under practical battery conditions, including high mass loading, fast charging, elevated temperature operation, and prolonged cycling, will be essential for bridging the gap between laboratory-scale discoveries and commercial applications.

## 6 Conclusions

DRX materials have emerged as promising Li-rich cathodes for next-generation rechargeable batteries. Compared with conventional Ni- and Co-rich layered oxides, DRX cathodes offer exceptional compositional flexibility and enable the incorporation of earth-abundant transition metals such as Fe, Ti, and Mn. Their potential for high capacity and low cost is therefore substantial. However, sluggish Li^+^ diffusion, poor intrinsic electronic conductivity, high carbon-additive requirements, voltage hysteresis, and interfacial degradation still hinder practical deployment. In this review, we summarized the mechanisms governing Li^+^ migration and electronic transport in DRX structures and emphasized the central trade-off between Li^+^ percolation and electronic conductivity. Increasing Li excess can enrich low-barrier 0-TM pathways, but excessive Li content dilutes redox-active TM networks and compromises electronic conduction. The selection of d^0^ transition-metal species, the regulation of SRO, and the introduction of favorable local ordering are therefore essential for optimizing transport kinetics and electrochemical performance. Looking forward, integrated strategies that combine non-equilibrium synthesis, δ-phase or partial-disorder engineering, electronic-conductivity optimization, electrolyte/interface stabilization, advanced *operando* characterization, and data-driven composition design will be required to realize practical DRX cathodes. Such advances may ultimately enable Co/Ni-lean or Co/Ni-free, low-cost, high-energy-density lithium-ion batteries with improved sustainability and long-term stability.

## Author contributions

Miaomiao Wang: draft preparation, reviewing & editing, visualization. Yuyang Zhang: visualization. Zhanhui Jia: conceptualization, reviewing. Hao Shen: reviewing. Wei Tang and Kai Chen: funding, supervision, reviewing.

## Conflicts of interest

The authors declare no conflict of interest.

## Data Availability

All data analysed in this review originate from previously published works and can be accessed *via* the cited references.
